# Phenology-aware collaborative retrieval of structural and physiological cotton traits from Sentinel-2 imagery using multi-task learning

**DOI:** 10.3389/fpls.2026.1928307

**Published:** 2026-08-25

**Authors:** Li Li, Jinjie Wang, Jianli Ding, Zhihong Liu, Aimaieraili Tuheti

**Affiliations:** 1 College of Geography and Remote Sensing Sciences, Xinjiang University, Urumqi, China; 2 Xinjiang Institute of Technology, Aksu, China; 3 Aksu Prefecture Agricultural Technology Extension Center, Aksu, China

**Keywords:** cotton, maximal information coefficient, multi-task regression, precision crop management, reliable observation window

## Abstract

Cotton leaf area index (LAI) and SPAD values characterize canopy structure and relative leaf chlorophyll status, respectively; however, most existing remote-sensing studies retrieve these two traits independently and pay limited attention to phenology-driven changes in their shared and target-specific spectral information. Using synchronous LAI and SPAD measurements collected from 109 sampling sites over eight observation periods in 2021, together with Sentinel-2 imagery, this study developed a phenology-dependent framework for the collaborative retrieval of LAI and SPAD. The maximal information coefficient (MIC) was used to identify sensitive spectral features for each observation period, after which three feature-input schemes and six regression algorithms were evaluated sequentially, with model performance evaluated using nested five-fold cross-validation grouped by sampling-site ID. The optimal model was subsequently applied to county-scale mapping and externally validated at the point scale using independent samples collected during T3, T4, and T6 in 2025. The results showed that (1) LAI, SPAD, and the composition of their sensitive spectral features exhibited pronounced phenological dependence, with red-edge features repeatedly selected across multiple periods, whereas features from other categories displayed stronger period specificity. (2) The combination of the period-specific union of sensitive features (MIC_union) and multi-task elastic net (MTE) achieved the best overall performance, yielding R^2^ values of 0.91 and 0.79 for LAI and SPAD, respectively, and a joint normalized error of 0.08 in the outer-loop cross-validation for 2021. In the 2025 validation, T3 retained the highest cross-year explanatory capacity, with R^2^ values of 0.61 and 0.53 and RMSE values of 0.23 and 3.04 for LAI and SPAD, respectively, whereas cross-year transferability was comparatively weak at T6. (3) Based on the period-specific evaluation for 2021 and the external validation for 2025, T3 was identified as the primary reliable window for collaborative LAI–SPAD mapping, with T4 serving as a supplementary window. This framework provides methodological support for the simultaneous remote-sensing characterization of cotton canopy structure and chlorophyll status during key growth stages, as well as for optimizing the timing of image acquisition and field surveys.

## 1 Introduction

The development of cotton canopy structure and the maintenance of leaf photosynthetic function jointly support dry matter accumulation and yield formation. Leaf area index (LAI), defined as the leaf area per unit ground area, is an important structural parameter for characterizing canopy development and its potential for light interception, transpiration, and gas exchange (Fang et al., 2019); SPAD values provide a nondestructive indicator of relative leaf chlorophyll status and are commonly used as an indirect measure of crop nitrogen and nutritional status (Wu et al., 1998; Uddling et al., 2007; Su et al., 2024). In crops such as maize, soybean, and rice, the same green LAI may correspond to a two- to threefold difference in canopy chlorophyll content, indicating that canopy structural quantity and chlorophyll status do not have a one-to-one relationship (Gitelson et al., 2022). Therefore, the simultaneous acquisition of LAI and SPAD information enables a more comprehensive assessment of cotton growth from the complementary perspectives of canopy structure and leaf physiology.

Optical remote sensing provides an effective means of acquiring crop structural and pigment-related information over large areas. The multiple red-edge bands available in Sentinel-2 imagery are highly sensitive to variations in both LAI and chlorophyll, providing a spectral basis for the simultaneous observation of these two traits (Delegido et al., 2011). Previous research has demonstrated the capability of Sentinel-2 data to estimate potato LAI, leaf chlorophyll content, and canopy chlorophyll content (Clevers et al., 2017). Regularized model inversion based on Landsat data has also been used for the joint retrieval of LAI and leaf chlorophyll content (Houborg et al., 2015). However, canopy reflectance is not a simple additive combination of structural and physiological signals. Reflectance responses in the red-edge and near-infrared regions are influenced not only by leaf area, leaf inclination angle, and canopy cover, but also by chlorophyll status and soil background. Failure to account for canopy structural and soil background effects may introduce substantial bias into chlorophyll retrieval (Wan et al., 2024). Consequently, a given spectral variable may contain information shared by LAI and SPAD or may respond predominantly to only one of the two targets. Single-target modeling and independent feature selection make it difficult to distinguish between these two types of information and cannot exploit the potential statistical association between LAI and SPAD.

The structural–physiological spectral relationships associated with LAI and SPAD do not remain constant throughout the growing season but exhibit pronounced phenological dependence. As crops transition from sparse canopies to canopy closure and subsequent senescence, vegetation cover, leaf area, pigment status, and background components change accordingly, thereby altering the relative contributions of structural, pigment-related, and background signals to canopy reflectance. Previous studies have shown that increasing canopy cover can cause the relationship between vegetation indices and chlorophyll content to shift gradually from linearity toward more pronounced nonlinearity (Qiao et al., 2022). Chlorophyll-sensitive bands and retrieval errors also differ markedly among growth stages, and a unified model developed using samples pooled across all periods often struggles to represent these stage-specific variations consistently (Delloye et al., 2018; Angel and McCabe, 2022; Gao et al., 2022a). However, the aforementioned phenology-related studies have primarily focused on a single chlorophyll trait and have not systematically distinguished between the shared sensitive information and target-specific information associated with LAI and SPAD, nor have they comprehensively evaluated how the stage-dependent variation in such information affects the reliability of joint dual-target retrieval.

The maximal information coefficient (MIC) can quantify various forms of linear and nonlinear statistical dependence between variables, providing an effective tool for identifying trait-sensitive spectral features across different growth stages (Reshef et al., 2011). In remote sensing of crop chlorophyll, MIC can identify nonlinear spectral responses under varying canopy-cover conditions more robustly than correlation coefficients, which primarily characterize linear relationships (Qiao et al., 2022). However, MIC measures only the pairwise association between an individual feature and a single target, and separate feature selection for LAI and SPAD cannot explicitly exploit the statistical relationship between the two targets. Multi-task learning jointly estimates multiple related targets within a unified framework, thereby exploiting information shared across tasks while preserving differences in their individual responses (Argyriou et al., 2008). This approach has been applied to the joint prediction of multiple image-based plant phenotypes and has shown potential for improving model generalization (Dobrescu et al., 2020); however, its application to the collaborative retrieval of crop structural and physiological traits from satellite remote sensing remains limited, particularly with respect to the integrated consideration of phenological variation, shared sensitive features, and target-specific features.

To address the research gaps identified above, this study combines period-specific MIC-based feature selection with MultiTaskElasticNet (MTE) to investigate phenological variations in the shared and target-specific spectral information associated with LAI and SPAD and to evaluate how these variations affect the applicability of joint dual-target retrieval. Focusing on representative cotton fields in Manas County, Xinjiang, this study integrates synchronous field measurements of LAI and SPAD collected during the T1–T8 observation periods in 2021 with Sentinel-2 time-series imagery to construct a phenology-dependent framework for collaborative LAI–SPAD retrieval. The specific objectives were to (1) analyze temporal variations in LAI, SPAD, and the composition of their sensitive spectral features; (2) evaluate the effects of different feature-organization strategies and regression algorithms on joint LAI–SPAD retrieval; (3) identify reliable observation windows based on out-of-sample predictions, conduct county-scale spatial mapping, and use independent samples from T3, T4, and T6 in 2025 to evaluate the point-scale cross-year applicability of the selected model during the corresponding periods.

## 2 Materials and methods

### 2.1 Study area

**Figure 1:**
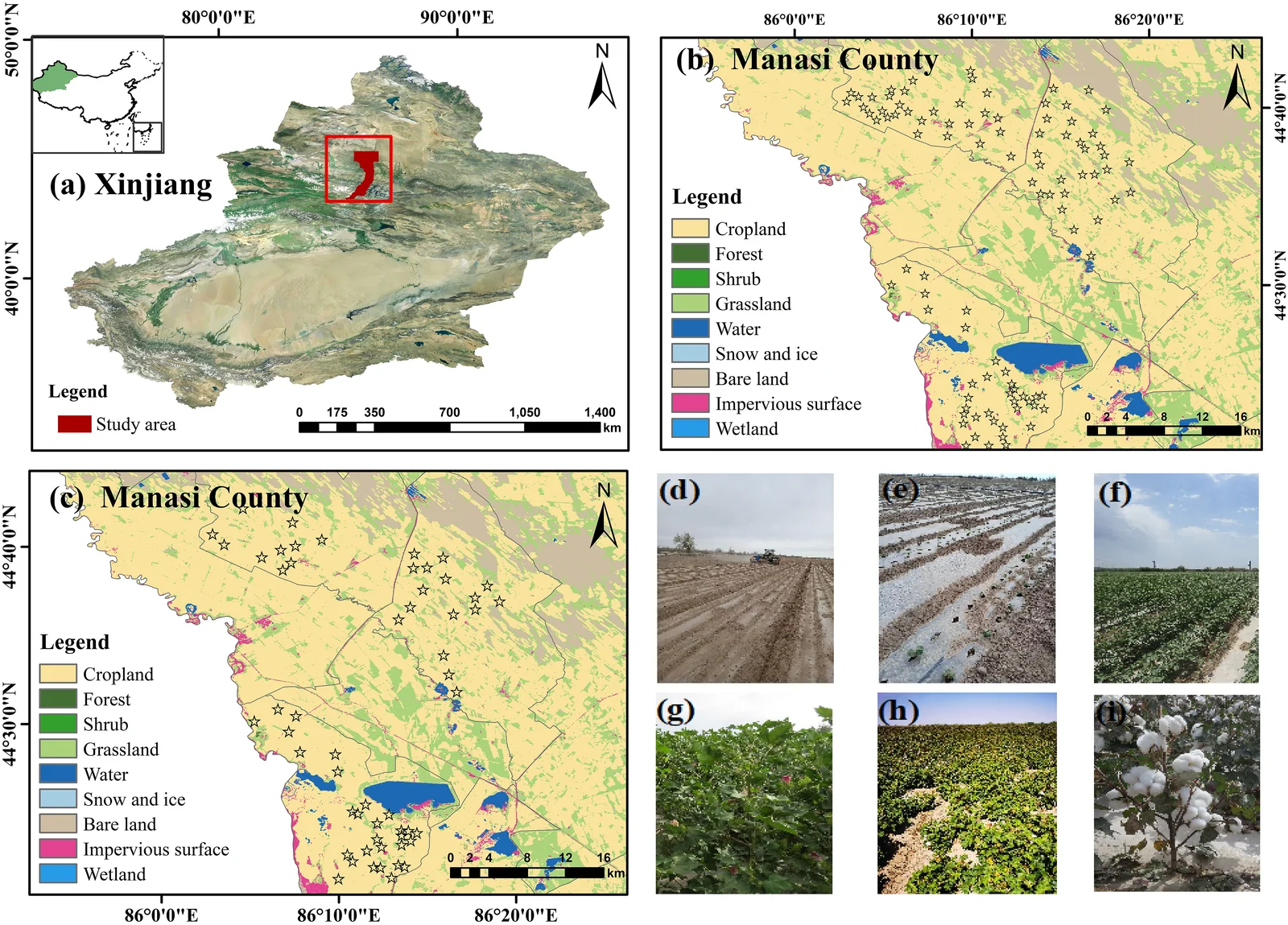
Study area and sampling locations: **(A)** location of the study area; **(B)** distribution of LAI and SPAD sampling sites in 2021; **(C)** distribution of LAI and SPAD sampling sites in 2025. **(D–I)** representative field photographs of cotton fields during different observation periods.

Field experiments were conducted in representative cotton fields in Manas County, Changji Hui Autonomous Prefecture, Xinjiang Uygur Autonomous Region, China (**Figure 1**). The study area is located on the northern piedmont of the Tianshan Mountains and the southern margin of the Junggar Basin (43°28′N–45°38′N, 85°34′E–86°43′E; average altitude: 522 m), representing an important oasis agricultural region in northern Xinjiang and a major production area for cotton and grain crops. The region has a temperate continental arid to semi-arid climate, characterized by abundant light and heat resources but substantial water limitation: the long-term mean annual precipitation is 100–200 mm, the mean annual temperature is approximately 7.5–8.2 °C, annual sunshine duration is about 2318–2732 h, and potential evapotranspiration reaches 1500–2100 mm (Shen et al., 2026).

**Figure 2:**
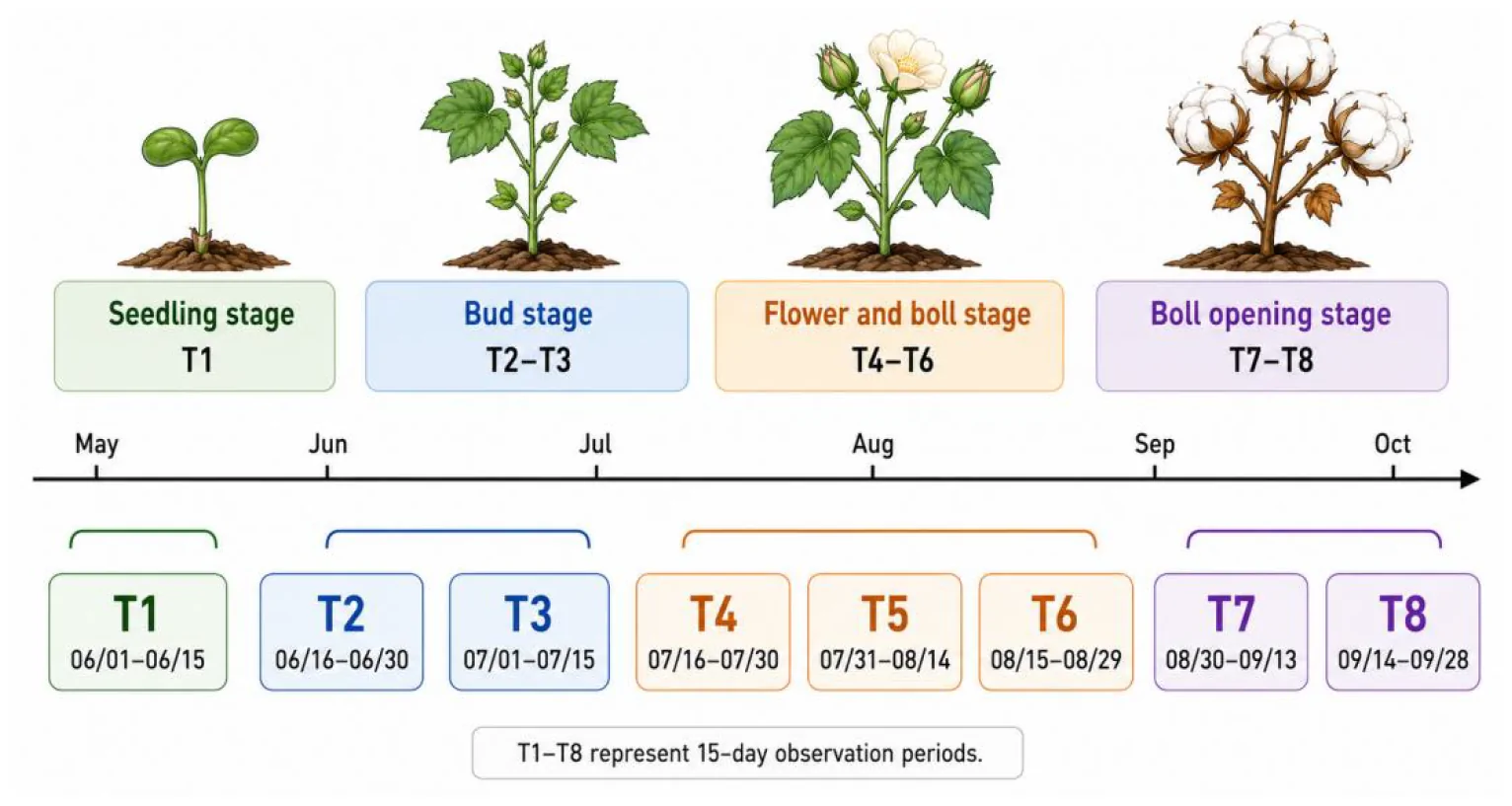
Schematic representation of the 15-day observation windows and corresponding cotton phenological stages.

Cotton is typically sown from mid-April to early May and enters the harvesting stage from late September to early October. Based on the growth and developmental characteristics of cotton, its growth cycle is generally divided into the emergence, seedling, squaring, flowering and boll-setting, and boll-opening stages (Wang et al., 2025). The correspondence between the T1–T8 observation windows and these phenological stages is illustrated in **Figure 2**.

### 2.2 Data acquisition

To ensure both the spatial representativeness of the samples and scale consistency between ground observations and Sentinel-2 pixels, 109 fixed 10 m × 10 m plots were established in representative cotton fields across the study area in 2021. The sampling design was intended to cover the spatial distribution of the major cotton-growing areas and observable differences in crop growth conditions, allowing the plots to represent the spatial heterogeneity among actual cotton fields. The 109 plots were repeatedly surveyed during the eight observation periods from T1 to T8, yielding a total of 872 plot–period records. A unique sampling point was designated within each plot, and its central coordinates were recorded using a handheld GPS receiver. Within each plot, a relatively homogeneous canopy area was selected while avoiding field boundaries, roads, irrigation canals, bare soil, and other land-cover features that could result in mixed pixels. LAI and SPAD were measured synchronously at five locations within each plot, comprising the four corners and the center. In 2025, 55 fixed plots were established using the same protocol and repeatedly surveyed at T3, T4, and T6, yielding 165 plot–period records.

#### 2.2.1 SPAD field measurements

Relative chlorophyll status was measured using a handheld SPAD-502 chlorophyll meter (Konica Minolta, Japan). At each of the five sampling positions within a plot—the four corners and the center—three fully expanded, normally growing functional leaves without disease, mechanical damage, or severe shading were selected, with one leaf sampled from each of the upper, middle, and lower canopy layers. Three SPAD readings were taken at different locations on the leaf blade of each selected leaf while avoiding the main vein, and the three readings were averaged to obtain a leaf-level SPAD value. The mean SPAD value of the three leaves was then calculated for each sampling position, and the mean across the five sampling positions was used as the plot-level SPAD value.

#### 2.2.2 LAI field measurements

LAI was measured using an LAI-2200C Plant Canopy Analyzer (LI-COR Biosciences, USA), with measurements conducted while minimizing exposure to direct sunlight. An above-canopy reference reading was first obtained in an unobstructed open area near the plot, after which the sensor was positioned near the base of the cotton plants to collect below-canopy readings; two consecutive below-canopy measurements were taken at each observation point, and their mean was used as the point-level LAI value. The LAI values from the five sampling positions were averaged to obtain the final plot-level LAI value.

#### 2.2.3 Satellite data

Sentinel-2 Level-2A surface reflectance products for the corresponding periods in 2021 and 2025 were acquired through the Google Earth Engine (GEE) platform. Continuous and non-overlapping 15-day temporal windows were defined according to the predetermined observation schedule, and each field campaign was assigned to the corresponding window that fully encompassed its start and end dates. Images with scene-level cloud cover exceeding 30% were first excluded, after which the QA60 band and Scene Classification Layer (SCL) were jointly used to mask clouds, cirrus clouds, cloud shadows, and other invalid pixels. Following image quality control, all valid observations within each temporal window were composited using a pixel-wise temporal median to generate a 15-day median composite for the corresponding observation period, hereafter referred to as W15. The nominal W15 windows and their corresponding phenological stages are illustrated in **Figure 2**, and the independent Sentinel-2 acquisition dates actually included in the composites are presented in **Supplementary Table S1**.

To evaluate the effect of temporal-window length on LAI and SPAD retrieval performance, afield-aligned 5-day median-compositing scheme (W5) was constructed based on the actual start and enddates of the eight field campaigns conducted in 2021. Each W5 window comprised the corresponding field-campaign end date and the four preceding complete calendar days, with no imagery acquired after the survey end date included. To ensure comparability, both W15 and W5 were evaluated using the same 853 valid plot–period records and identical nested five-fold partitions grouped by sampling-site ID, feature-selection procedures, hyperparameter-optimization strategies, and evaluation workflows. W5 was used exclusively for the temporal-window sensitivity analysis, and the corresponding windows and image-acquisition dates are provided in **Supplementary Table S3**.

Ten Sentinel-2 spectral bands were selected, and the 20 m bands were resampled to 10 m using bilinear interpolation and aligned with the native 10 m band grid. Cotton fields were masked using a 10 m cotton distribution product for Xinjiang, with pixels having a cotton probability of ≥0.5 classified as cotton pixels (Kang et al., 2023). After temporal compositing, resampling, and spectral-feature calculation, the center coordinates of the field plots were imported into GEE and spatially matched to the Sentinel-2–10 m pixel grid. For each observation period, the values of the pixel containing the center coordinate of each plot were extracted for all spectral bands and vegetation indices. Only pixels retaining valid values after QA60/SCL masking and cotton-field masking were included. The extracted spectral features were then matched with the corresponding plot-level LAI and SPAD observations according to sampling-site ID and observation period. All image preprocessing, spectral-feature calculation, and pixel-value extraction were conducted in GEE. The number of independent acquisition dates and the proportion of valid matches for each observation period are presented in **Table 1**.

**Table 1:** Sentinel-2 image availability and valid observations within 15-day composite windows for 2021 county-scale mapping and 2025 point-scale external validation.

Year	Period	Cloud-filtered S2 records	Cloud-filtered unique dates	Valid county cotton pixels	Valid matched samples	Valid coverage/matching rate (%)
2021	T1	24	5	13,929,336	109/109	100.00
2021	T2	8	3	7,222,650	109/109	51.85
2021	T3	27	5	13,929,336	109/109	100.00
2021	T4	17	3	13,882,344	109/109	99.66
2021	T5	30	5	13,929,336	109/109	100.00
2021	T6	25	5	13,905,327	109/109	99.83
2021	T7	34	5	13,929,336	109/109	100.00
2021	T8	34	6	13,929,336	109/109	100.00
2025	T3	27	5	N/A	55/55	100.00
2025	T4	26	5	N/A	55/55	100.00
2025	T6	31	6	N/A	55/55	100.00

Cloud-filtered S2 records and unique dates denote the numbers of retained Sentinel-2 images and independent acquisition dates, respectively. Valid county cotton pixels represent cotton-mask pixels with valid reflectance values in each W15 composite. For 2021, the final column reports county cotton-pixel coverage; for 2025, it reports the proportion of validation samples successfully matched with Sentinel-2 features. County-scale pixel statistics were not calculated for 2025.

### 2.3 Sentinel-2 spectral feature construction

Based on Sentinel-2 Level-2A surface reflectance products, this study selected 10 original bands (B2, B3, B4, B5, B6, B7, B8, B8A, B11, and B12) and 20 vegetation indices, generating a total of 30 candidate spectral features for the collaborative retrieval of cotton LAI and SPAD (**Table 2**). The selected features cover the visible, red-edge, near-infrared, and shortwave-infrared regions, providing information related to chlorophyll absorption, red-edge displacement, canopy near-infrared scattering, water status, and soil background. Among these features, red-edge variables have been widely used for estimating crop chlorophyll, nitrogen status, and LAI, whereas near-infrared and shortwave-infrared features can provide complementary information on canopy structure, water status, and background variability (Gao, 1996; Ma et al., 2025; Pancorbo et al., 2025). Fractional vegetation cover (FVC) was calculated using an NDVI-based dimidiate pixel model (Gutman and Ignatov, 1998). For each observation period, *NDVI_soil_* and *NDVI_veg_* were defined as the 5th and 95th percentiles, respectively, of the NDVI distribution of valid cotton-field pixels in the W15 composite, and FVC values were constrained to the range of 0–1.

**Table 2:** Vegetation indices calculated from Sentinel-2 images in this study.

Component	Variable	Formulation	References
Visible	B2, B3, B4	–	–
Red-edge	B5, B6, B7, B8A	–	–
	NDRE	$\text{NDRE}=\frac{\text{NIR}-{\text{RE}}_{1}}{\text{NIR}+{\text{RE}}_{1}}$	(Clevers and Gitelson, 2013)
	CIrededge	${\text{CI}}_{\text{rededge}}=\frac{\text{NIR}}{{\text{RE}}_{1}}-1$	(Gitelson et al., 2003)
	MTCI	$\text{MTCI}=\frac{{\text{RE}}_{2}-{\text{RE}}_{1}}{{\text{RE}}_{1}-\text{R}}$	(Dash and Curran, 2004)
	S2REP	$S2REP=705+35\times \frac{\frac{\text{R}+{\text{RE}}_{3}}{2}-{\text{RE}}_{1}}{{\text{RE}}_{2}-{\text{RE}}_{1}}$	(Frampton et al., 2013)
NIR–structure	B8	–	–
	NDVI	$\text{NDVI}=\frac{\text{NIR}-\text{R}}{\text{NIR}+\text{R}}$	(Tucker, 1979)
	EVI	$\text{EVI}=2.5\times \frac{\text{NIR}-\text{R}}{\text{NIR}+6R-7.5\text{B}+1}$	(Huete et al., 2002)
	SAVI	$\text{SAVI}=\frac{\left(1+\text{L})(\text{NIR}-\text{R}\right)}{\text{NIR}+\text{R}+\text{L}}\text{, L}=0.5$	(Huete, 1988)
	OSAVI	$\text{OSAVI}=\frac{1.16(\text{NIR}-\text{R})}{\text{NIR}+\text{R}+0.16}$	(Rondeaux et al., 1996)
	MSAVI	$\text{MSAVI}=\frac{2\text{NIR}+1-\sqrt{{\left(2\text{NIR}+1\right)}^{2}-8(\text{NIR}-\text{R})}}{2}$	(Qi et al., 1994)
	RDVI	$\text{RDVI}=\frac{\text{NIR}-\text{R}}{\sqrt{\text{NIR}+\text{R}}}$	(Roujean and Breon, 1995)
	MSR	$\text{MSR}=\frac{\frac{\text{NIR}}{\text{R}}-1}{\sqrt{\frac{\text{NIR}}{\text{R}}+1}}$	(Chen, 1996)
	NIR_v_	${\text{NIR}}_{\text{v}}=\text{NDVI}\times \text{NIR}$	(Badgley et al., 2017)
	GNDVI	$\text{GNDVI}=\frac{\text{NIR}-\text{G}}{\text{NIR}+\text{G}}$	(Gitelson et al., 1996)
	FVC	$\text{FVC}=\frac{\text{NDVI}-{\text{NDVI}}_{\text{soil}}}{{\text{NDVI}}_{\text{veg}}-{\text{NDVI}}_{\text{soil}}}$	(Gutman and Ignatov, 1998)
SWIR–moisture	B11, B12	–	–
	NDMI	$\text{NDMI}=\frac{\text{NIR}-{\text{SWIR}}_{1}}{\text{NIR}+{\text{SWIR}}_{1}}$	(Gao, 1996)
	NDTI	$\text{NDTI}=\frac{{\text{SWIR}}_{1}-{\text{SWIR}}_{2}}{{\text{SWIR}}_{1}+{\text{SWIR}}_{2}}$	(Van Deventer et al., 1997)
Chlorophyll	CIgreen	${\text{CI}}_{\text{green}}=\frac{\text{NIR}}{\text{G}}-1$	(Gitelson et al., 2003)
	MCARI	$\text{MCARI}=[({\text{RE}}_{1}-\text{R})-0.2({\text{RE}}_{1}-\text{G})]\times \frac{{\text{RE}}_{1}}{\text{R}}$	(Daughtry et al., 2000)
	TCARI	$\text{TCARI}=3[({\text{RE}}_{1}-\text{R})-0.2({\text{RE}}_{1}-\text{G})\times \frac{{\text{RE}}_{1}}{\text{R}}]$	(Haboudane et al., 2002)
	TCARI/OSAVI	$\text{TCARI}/\text{OSAVI}=\frac{\text{TCARI}}{\text{OSAVI}}$	(Haboudane et al., 2002)

### 2.4 Model construction

#### 2.4.1 Data partitioning and preprocessing

The 2021 dataset was used for model development, model comparison, and internal evaluation of out-of-sample predictive performance. Internal evaluation was conducted using nested five-fold GroupKFold cross-validation, with sampling-site ID specified as the grouping variable. The five outer folds were used to evaluate out-of-sample predictive performance, whereas an additional five-fold cross-validation was performed within each outer training set for hyperparameter optimization. All records from the same sampling site across different observation periods were consistently assigned to the same fold, preventing repeated measurements from an identical spatial location from entering both the training and validation sets. All continuous input features were standardized using Z-score normalization (Equation 1):

(1) $$\tilde{x}=\frac{x-\mu_{train}}{\sigma_{train}}$$

Here, *μ_train_* and *σ_train_* denote the mean and standard deviation of the training set, respectively. During inner-loop hyperparameter optimization, the standardization parameters were estimated exclusively from the corresponding inner training fold and then applied to the inner validation fold; during outer-loop model evaluation, these parameters were re-estimated using the complete outer training set and subsequently applied to the corresponding outer validation set.

The 2025 samples collected at T3, T4, and T6 were used exclusively for independent external validation at the point scale and were not involved in feature selection, model selection, hyperparameter optimization, or the estimation of standardization parameters. The period-specific models selected using the 2021 data and fitted to the complete 2021 dataset were directly applied to the 2025 samples without retraining, parameter adjustment, or bias correction during validation.

#### 2.4.2 Period-specific MIC feature selection and cross-period overlap analysis

Because canopy structure, chlorophyll status, and their spectral response relationships vary across cotton growth stages, MIC-based feature selection was conducted separately for each observation period from T1 to T8, with LAI and SPAD used independently as target variables to identify sensitive spectral features. To prevent information leakage during feature selection, MIC calculation, threshold-based screening, and redundancy removal were performed exclusively using the current training data in every inner- and outer-loop iteration of the nested cross-validation procedure; the feature set identified from the training data was subsequently applied to the corresponding validation data, which were not involved in either MIC calculation or feature selection. For period *t*, with LAI and SPAD separately treated as the target variable *Y*, MIC between each candidate feature X_j_ and *Y* is defined by Equation 2:

(2) $$MIC\left(X_{j},Y\right)=\underset{G\in G}{max}\frac{I\left(X_{j},Y|G\right)}{{log}_{2}min\left(\left|G_{x}\left|,\right|G_{y}\right|\right)}$$

Here, *G* denotes the set of two-dimensional grid partitions satisfying the search constraints, *G_x_* and *G_y_* represent the numbers of grid divisions along the *X_j_* and *Y* directions, respectively, and *I_G_(X_j_, Y)* denotes the mutual information calculated under grid *G*. MIC values range from 0 to 1, with larger values indicating stronger dependence between the candidate feature and the target variable. MIC values were computed in Python 3.10.20 using the MINE implementation provided by minepy 1.2.6. The approximation parameters were fixed at alpha = 0.6 and c = 15, with the est parameter set to “mic_approx”, for all target variables, observation periods, and cross-validation folds. The same implementation and parameter settings were used for both training-fold feature selection and final full-sample feature analysis.

The feature-selection procedure comprised two steps. First, preliminary screening was performed separately for LAI and SPAD, and features with MIC ≥ 0.30 were retained as sensitive features for the corresponding target variable. Second, redundant features were removed separately within the LAI-sensitive and SPAD-sensitive feature sets. Candidate features were first ranked in descending order according to their MIC values with the target variable; the feature with the highest remaining MIC was retained sequentially, and subsequent features exhibiting a pairwise MIC ≥ 0.90 with the retained feature were removed until no highly redundant feature combinations remained (Li et al., 2024).

Three input schemes were constructed based on the LAI-sensitive and SPAD-sensitive feature sets identified separately for each observation period. The full feature set (All_features) contained all 30 candidate spectral variables; the shared sensitive feature set (MIC_shared) was defined as the intersection of the LAI-sensitive and SPAD-sensitive feature sets and represented spectral information associated with both targets; the union of sensitive features (MIC_union) comprised the union of the two target-specific sensitive feature sets, thereby retaining both shared sensitive features and target-specific features exhibiting a strong dependency on only one of the two targets. The three schemes were used to compare the effects of different feature-organization strategies on collaborative LAI–SPAD retrieval performance.

**Figure 3:**
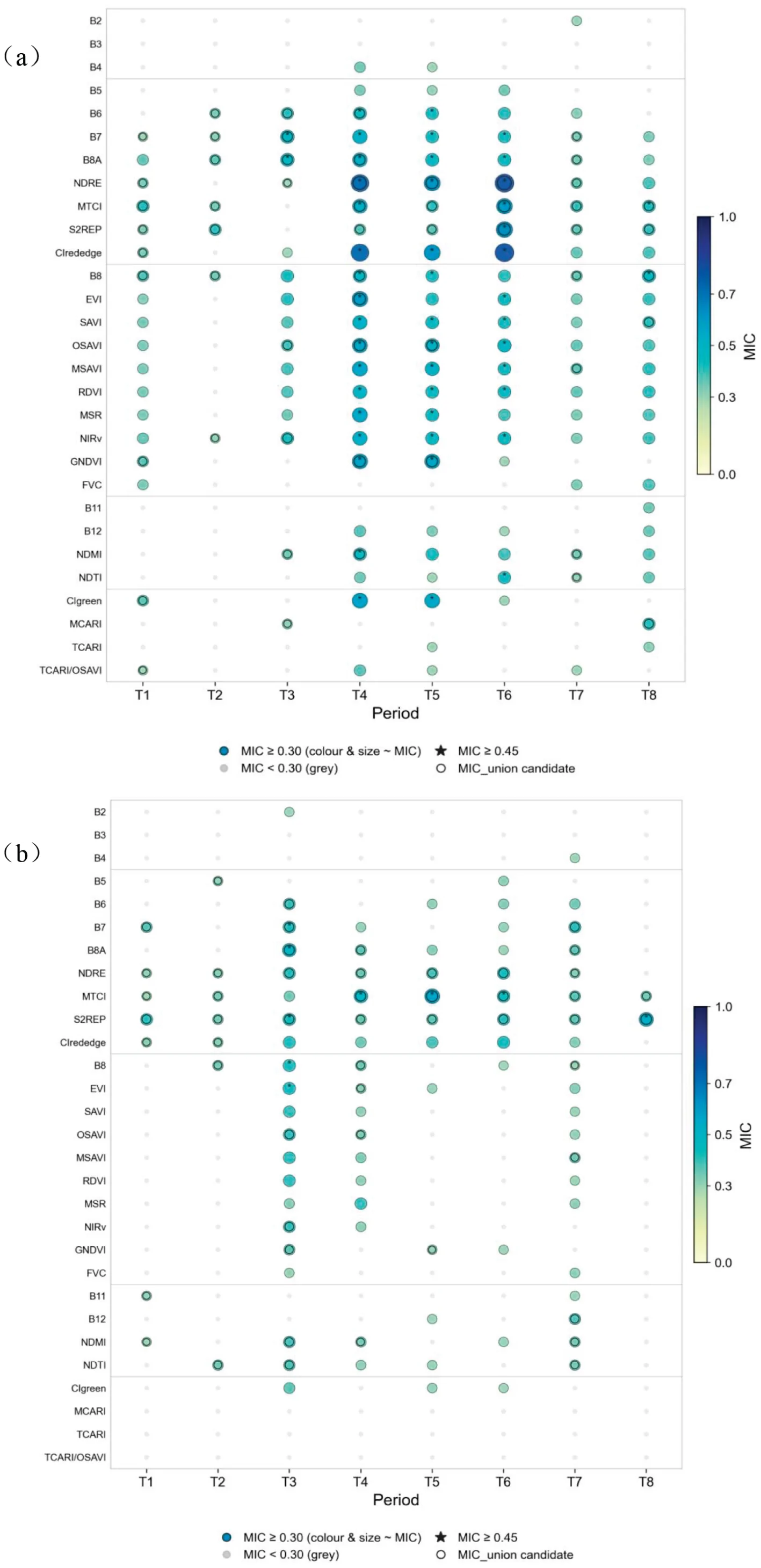
Period-specific maximum information coefficient (MIC) values between individual spectral features and LAI and SPAD from T1 to T8: **(a)** LAI and **(b)** SPAD. Each row represents a Sentinel-2 band or vegetation index, and each column represents an observation period. Point color and size indicate the MIC value. Grey points indicate MIC < 0.30, colored points indicate MIC ≥ 0.30, stars indicate MIC ≥ 0.45, and open circles identify features retained in the final period-specific MIC_union feature sets after screening and redundancy removal.

**Figure 4:**
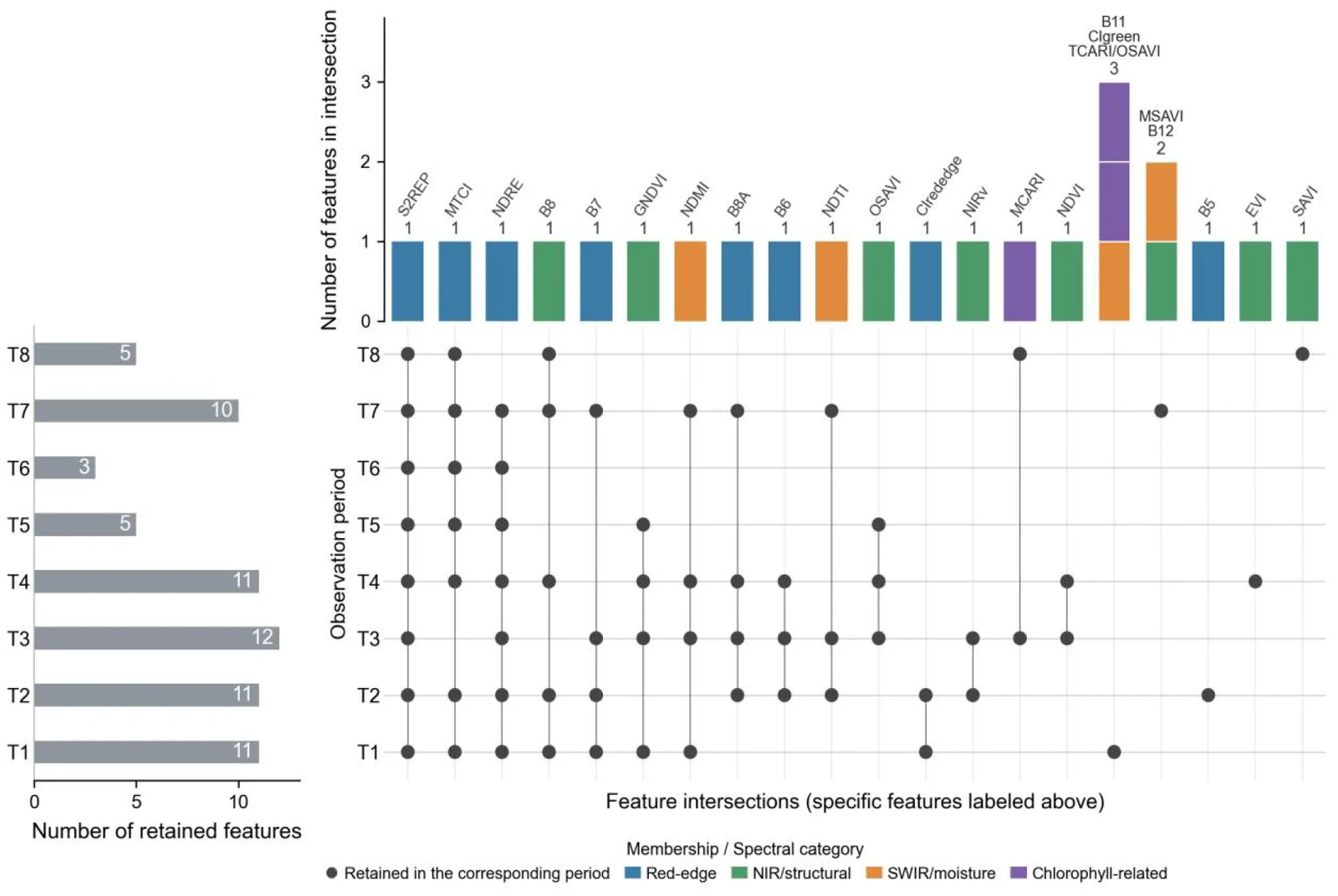
Temporal overlap of period-specific MIC_union features. The horizontal bars show the number of features retained in each observation period. Filled dots indicate the periods in which each feature or feature group was retained, while the upper bars show the corresponding intersection sizes. Bar colors indicate spectral categories.

After completing cross-validation and model comparison, the final feature set for each observation period was re-determined using all 2021 samples according to the same selection rules and was subsequently used for feature interpretation and final spatial mapping. **Figure 3**, **Figure 4**, and **Supplementary Table S6** were all based on the feature-selection results obtained from the complete dataset. An UpSet plot was further used to characterize the cross-period overlap patterns of the final MIC_union feature sets across T1–T8, where the set size indicates the total number of retained features in each period, and the intersection size indicates the number of features shared among the corresponding combination of periods. The full-sample feature analysis described above was used exclusively for result interpretation and spatial mapping and was not involved in out-of-fold (OOF) performance calculation, hyperparameter optimization, or model comparison.

#### 2.4.3 Comparison of joint modeling strategies

To determine the final model configuration, the alternative feature schemes and regression algorithms were first compared to identify the optimal feature–model combination; this combination was then fixed, and different phenological modeling strategies were further evaluated. Period-specific modeling involved constructing an independent dual-output model for each period from T1 to T8 using the corresponding period-specific MIC_union feature set; phenological-group modeling involved fitting a shared model separately for the seedling, squaring, flowering and boll-setting, and boll-opening stages using the intersection of the MIC_union feature sets across periods within each group; global modeling pooled samples from all periods to fit a single unified model using the intersection of the MIC_union feature sets across T1–T8. In every inner- and outer-loop iteration of nested cross-validation, the period-specific feature sets and their intersections described above were determined exclusively from the current training fold and then applied to the corresponding validation fold.

#### 2.4.4 Regression algorithms and parameter settings

Six regression algorithms were compared, including MultiTaskElasticNet (MTE), random forest (RF), partial least squares regression 2 (PLS2), extreme gradient boosting (XGBoost), Light Gradient Boosting Machine (LightGBM), and radial basis function support vector regression (SVR-RBF). MTE and PLS2 are native multi-output models for joint LAI–SPAD regression, while RF directly supports multi-output regression within the scikit-learn framework; XGBoost, LightGBM, and SVR-RBF were wrapped using MultiOutputRegressor to enable dual-target prediction of LAI and SPAD. All regression-modeling procedures were implemented in Python 3.12.7 using scikit-learn 1.5.1, xgboost 1.7.6, and lightgbm 4.6.0.

Hyperparameter optimization was performed within each outer training set. Grid search was usedfor MTE, RF, PLS2, and SVR-RBF, whereas random search was applied to XGBoost and LightGBM, with 50randomly sampled candidate hyperparameter combinations evaluated separately for each of the latter two models. All candidate models were compared using the same inner five-fold grouped cross-validation splits. The implementation, fixed settings, search method, and complete hyperparameter search space for each model are provided in **Supplementary Table S7**.

To eliminate the influence of differences in the numerical scales of LAI and SPAD on parameter selection, the joint normalized error (JNE) was used to evaluate the candidate hyperparameter combinations. For the *k*th inner validation fold, the RMSE values for LAI and SPAD were calculated separately and normalized by the ranges of the corresponding target variables in the associated inner training fold (Equation 3):

(3) $$JNE_{k}=\frac{1}{2}\left(\frac{RMSE_{LAI,k}}{LAI_{range,train,k}}+\frac{RMSE_{SPAD,k}}{SPAD_{range,train,k}}\right)$$

*LAI_range,train,k_* and *SPAD_range,train,k_* denote the differences between the maximum and minimum observed LAI and SPAD values, respectively, in the *kth* inner training fold. The optimization score for each candidate hyperparameter combination was calculated as the mean JNE across the five inner validation folds, and the combination with the lowest mean JNE was selected as the optimal configuration for the corresponding outer training set. All normalization ranges, MIC-based feature sets, and standardization parameters were determined exclusively from the corresponding training folds.

After comparing the feature-selection strategies, regression algorithms, and modeling strategies, a unified final hyperparameter-selection procedure was conducted using the complete 2021 modeling dataset. This procedure used five-fold GroupKFold partitioning with sampling-site ID as the grouping variable, the same hyperparameter search spaces and JNE optimization criterion as those used during model development, and aggregated the dual-target prediction errors of the T1–T8 samples within each validation fold. The single optimal hyperparameter configuration identified for each algorithm was then applied consistently to the period-specific models for T1–T8. Subsequently, the final MIC_union feature set and standardization parameters were re-determined separately for each period using all corresponding 2021 samples, and the period-specific models were fitted under the unified hyperparameter configuration.

#### 2.4.5 Model evaluation

The internal generalization performance of the models was evaluated using out-of-fold (OOF) predictions generated through the outer five-fold GroupKFold cross-validation. Each observation received exactly one prediction in the outer validation fold for which it had not been used in MIC-based feature selection, estimation of standardization parameters, hyperparameter optimization, or model training. Overall performance was calculated from all OOF predictions pooled across the five outer validation folds, whereas period-specific performance was calculated separately using the corresponding OOF predictions for T1–T8. Identical outer data partitions were used for all feature-input schemes, regression algorithms, and modeling strategies.

Internal model performance was evaluated using the coefficient of determination (R^2^; Equation 4), root mean square error (RMSE; Equation 5), mean absolute error (MAE; Equation 6), and joint normalized error (JNE). The fold-specific JNE used for inner-loop hyperparameter selection was calculated according to Equation 3. For final OOF performance aggregation, the same normalized-error structure was applied to the pooled OOF predictions, with normalization based on the overall observed ranges of LAI and SPAD across all 2021 modeling samples. For the 2025 point-scale external validation, the mean bias error (MBE; Equation 7) was additionally calculated.

(4) $$R^{2}=1-\frac{\sum_{i=1}^{n}{\left(O_{i}-P_{i}\right)}^{2}}{\sum_{i=1}^{n}{\left(O_{i}-\bar{O}\right)}^{2}}$$

(5) $$RMSE=\sqrt{\frac{1}{n}\sum_{i=1}^{n}{\left(O_{i}-P_{i}\right)}^{2}}$$

(6) $$MAE=\frac{1}{n}\sum_{i=1}^{n}|O_{i}-P_{i}|$$

(7) $$MBE=\frac{1}{n}\sum_{i=1}^{n}(P_{i}-O_{i})$$

Here, *O_i_* and *P_i_* denote the observed and predicted values, respectively, $\bar{O}$ represents the mean of the observed values, and *n* is the number of samples. A positive MBE indicates overall overestimation, whereas a negative MBE indicates overall underestimation; an absolute value closer to zero represents a smaller systematic bias.

## 3 Results

### 3.1 Phenological patterns of LAI and SPAD

**Figure 5:**
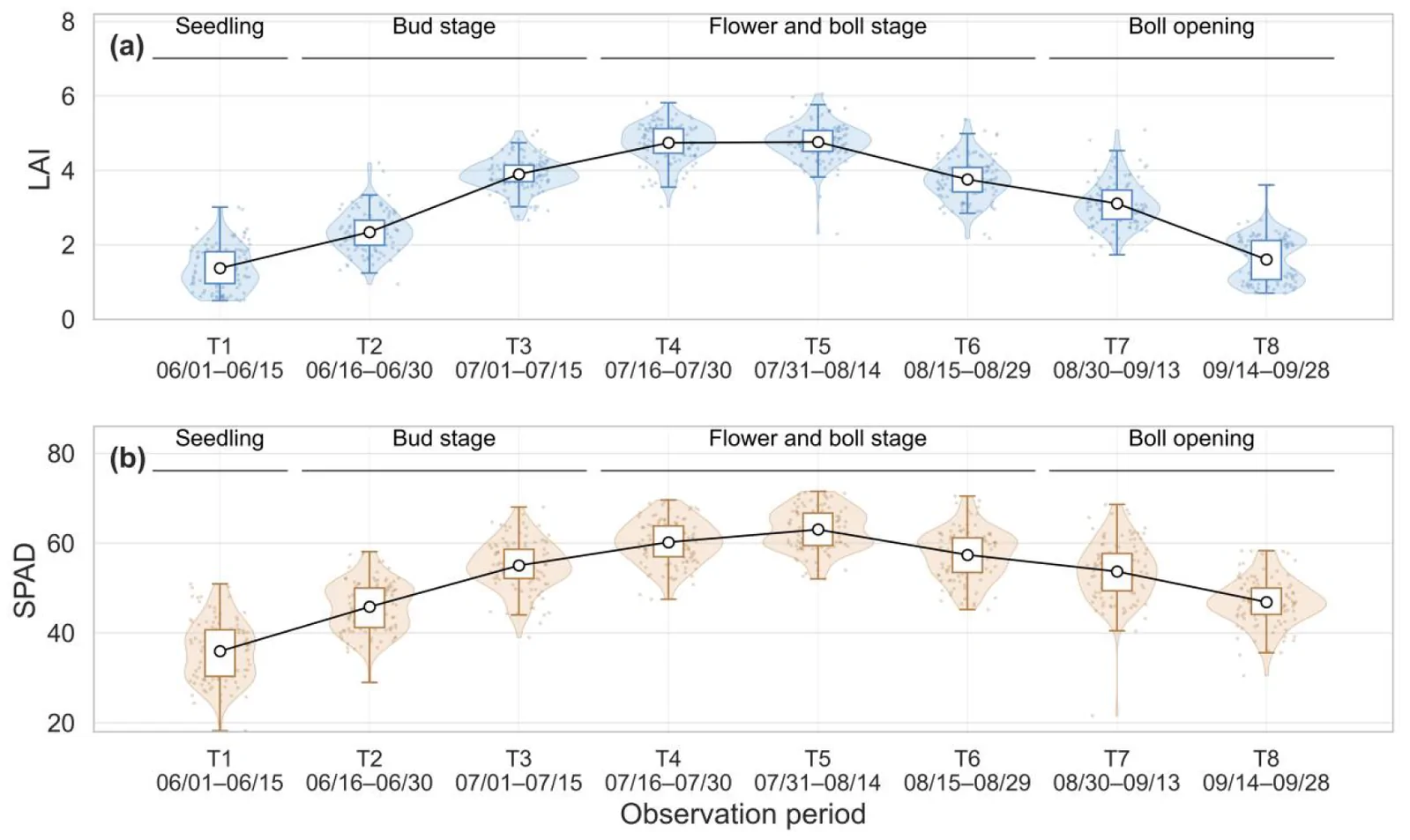
Phenology-dependent distributions and temporal variation of **(A)** LAI and **(B)** SPAD across eight observation periods. Violin plots show the distributions of individual observations, embedded boxplots indicate the medians and interquartile ranges, and black lines with open circles represent the period-specific mean values. The horizontal lines above each panel indicate the corresponding cotton phenological stages.

LAI exhibited a pronounced increase followed by a decrease across the eight observation periods (**Figure 5A**). LAI increased continuously from T1 to T4, remained at its highest level during T4–T5, and then gradually decreased from T6 onward. SPAD also showed an initial increase followed by a decrease, although its variation among observation periods was relatively moderate (**Figure 5B**). SPAD gradually increased from T1 to T5, reached its maximum at T5, and then decreased from T6 onward. Overall, both LAI and SPAD remained at relatively high levels during the middle stage of cotton growth, but their peak dynamics were not fully synchronized: LAI formed a high-value plateau during T4–T5, whereas SPAD reached its maximum at T5.

### 3.2 MIC-based nonlinear spectral sensitivity and feature dynamics of LAI and SPAD

The MIC-based feature sensitivity analysis showed that the MIC values, numbers of sensitive features, and feature compositions associated with LAI and SPAD differed markedly across T1–T8 (**Figure 3**). For LAI, features with relatively high MIC values were concentrated mainly among red-edge, near-infrared/structural, and selected moisture-related features, with a comparatively larger number of high-MIC features observed during T4–T6 (**Figure 3A**). Among the red-edge-related features, B7, B8A, NDRE, MTCI, S2REP, and CIrededge met or exceeded the MIC screening threshold during multiple periods; among the near-infrared/structural features, B8, EVI, SAVI, OSAVI, MSAVI, RDVI, MSR, NIR_v_, and GNDVI exhibited moderate or high MIC values mainly during T3–T7. Features such as NDMI, NDTI, and CIgreen also reached the screening threshold during certain periods. In contrast, visible bands such as B2–B4 had MIC values below 0.30 during most observation periods.

The MIC patterns associated with SPAD also varied across observation periods, but the number and composition of its sensitive features differed from those of LAI (**Figure 3B**). The candidate features associated with SPAD were concentrated mainly among red-edge features and selected near-infrared/structural and moisture-related variables. A relatively large number of features reached the MIC screening threshold at T3, including B6, B7, B8A, NDRE, MTCI, S2REP, CIrededge, and multiple near-infrared/structural indices. MTCI, S2REP, and NDRE repeatedly reached the screening threshold across multiple observation periods; among them, MTCI maintained relatively high MIC values at T5 and T6, whereas S2REP exhibited a high MIC value at T8. Shortwave-infrared or moisture-related features, including B11, B12, NDMI, and NDTI, reached the screening threshold only during certain periods. MCARI, TCARI, and TCARI/OSAVI had MIC values below 0.30 during most observation periods.

The size and overlap relationships of the final MIC_union feature sets across different periods are shown in **Figure 4**. The number of retained features ranged from 3 to 12 across periods, with T3 having the largest set (12 features) and T6 the smallest (3 features). The UpSet analysis indicated that most features exhibited clear period specificity and were retained in only one or a few periods; meanwhile, S2REP was retained across all periods from T1 to T8, and MTCI and NDRE also recurred in multiple periods.

Regarding the specific feature composition, 23 of the original 30 candidate spectral variableswere included in the final MIC_union feature set during at least one observation period. These included eight red-edge features, eight near-infrared/structural features, four shortwave-infrared/moisture-related features, and three chlorophyll-related features. S2REP was retained throughout all periods from T1 to T8, MTCI was retained in seven periods except T3, and NDRE was retained in seven periods from T1 to T7. Among the near-infrared/structural features, B8 was retained in five periods; B7, B8A, and GNDVI were each retained in four periods; OSAVI was retained in three periods; NIR_v_ and NDVI were each retained in two periods; and EVI, MSAVI, and SAVI were retained in only one period each. Among the shortwave-infrared/moisture-related features, NDMI was retained in four periods, NDTI in three periods, and B11 and B12 only at T1 and T7, respectively. According to their membership in the LAI- and SPAD-sensitive feature sets, the candidate features in the MIC_union feature network were further classified into three categories: LAI-sensitive, SPAD-sensitive, and shared features (**Supplementary Figure 1**).

### 3.3 Stepwise comparison of feature schemes, regression algorithms, and modeling strategies

Following the sequential comparison design, the three feature-input schemes—All_features, MIC_shared, and MIC_union—were first compared within the period-specific MTE framework (**Table 3**). MIC_union achieved the lowest JNE and the best overall dual-target predictive performance, with R^2^, RMSE, and MAE values of 0.91, 0.40, and 0.29 for LAI, respectively, and corresponding values of 0.79, 4.50, and 3.52 for SPAD. In comparison, both MIC_shared and All_features showed reduced joint predictive performance.

**Table 3:** Ablation comparison of MIC-based feature strategies under the period-specific MTE model.

Feature scheme	LAI R^2^	LAI RMSE	LAI MAE	SPAD R^2^	SPAD RMSE	SPAD MAE	JNE
MIC_union	0.91	0.40	0.29	0.79	4.50	3.52	0.08
MIC_shared	0.88	0.44	0.32	0.75	5.00	3.93	0.09
All_features	0.90	0.42	0.29	0.69	5.56	3.53	0.09

To examine whether the results of the feature-scheme comparison depended on MTE, additionalsensitivity analyses were conducted using PLS2 as a representative linear multi-output model and RFas a representative nonlinear multi-output model (**Supplementary Table S8**). In the PLS2 model, MIC_union achieved the lowest JNE and the lowest absolute errors for both LAI and SPAD; in the RF model, MIC_union likewise yielded the lowest JNE. Based on the combined results from MTE and the PLS2 and RF sensitivity analyses, MIC_union was selected as the common feature-input scheme for the subsequent comparison of the six regression algorithms.

Under the same MIC_union feature scheme and outer GroupKFold partitions, the six regression algorithms exhibited different levels of dual-target predictive performance (**Table 4**). MTE achieved the best overall performance. PLS2 showed SPAD predictive performance comparable to that of MTE, but produced slightly higher errors for LAI, whereas RF ranked second in overall performance. XGBoost and LightGBM both achieved a JNE of 0.09, whereas SVR-RBF showed the lowest overall performance. Therefore, MTE was selected as the regression algorithm for the subsequent comparison of modeling strategies and final spatial mapping.

**Table 4:** Performance comparison of regression models using the MIC_union feature set under period-specific modeling.

Model	LAI R^2^	LAI RMSE	LAI MAE	SPAD R^2^	SPAD RMSE	SPAD MAE	JNE
MTE	0.91	0.40	0.29	0.79	4.50	3.52	0.08
Random Forest	0.89	0.43	0.32	0.77	4.82	3.71	0.08
PLS2	0.90	0.42	0.31	0.79	4.51	3.56	0.08
XGBoost	0.89	0.44	0.32	0.76	4.88	3.76	0.09
LightGBM	0.89	0.44	0.33	0.76	4.92	3.86	0.09
SVR-RBF	0.78	0.64	0.44	0.71	5.37	4.03	0.11

The comparison of different phenological modeling strategies further demonstrated that collaborative LAI–SPAD retrieval exhibited clear phenological dependence (**Table 5**). Under the MIC_union + MTE framework, period-specific modeling achieved the best performance for LAI estimation. For SPAD, period-specific modeling and phenology-grouped modeling both achieved an R^2^ of 0.79, with similar absolute errors and identical JNE values of 0.08. In contrast, global modeling showed weaker overall performance, with the R^2^ values for LAI and SPAD decreasing to 0.87 and 0.75, respectively.

**Table 5:** Performance comparison of phenology-dependent modeling strategies for joint LAI–SPAD retrieval.

Strategy	LAI R^2^	LAI RMSE	LAI MAE	SPAD R^2^	SPAD RMSE	SPAD MAE	JNE
Period-specific	0.91	0.40	0.29	0.79	4.50	3.52	0.08
Phenology-grouped	0.89	0.45	0.35	0.79	4.49	3.49	0.08
Global	0.87	0.49	0.39	0.75	4.95	3.91	0.09

### 3.4 Period-specific performance and reliable mapping windows of the optimized model

**Figure 6:**
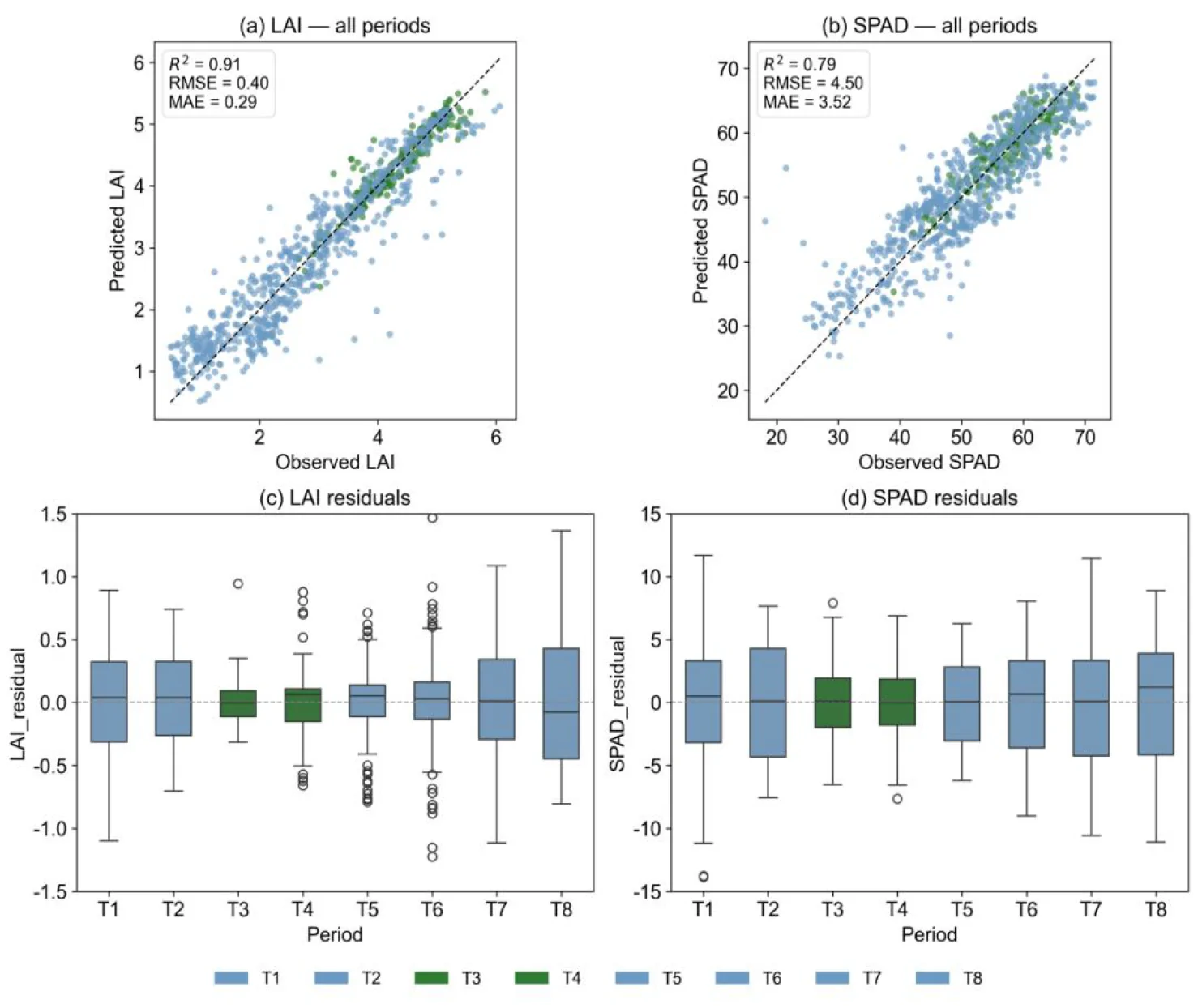
Overall out-of-fold prediction performance and period-specific residual distributions of the optimized model. Panels show **(a)** observed versus predicted LAI across all periods, **(b)** observed versus predicted SPAD across all periods, **(c)** LAI residual distributions across T1–T8, and **(d)** SPAD residual distributions across T1–T8.

The optimized MIC_union + MTE model exhibited strong overall predictive performance (**Figures 6A, B**). The predicted values were generally distributed close to the 1:1 line, indicating that the model effectively captured the major spatiotemporal variations in cotton canopy structure and chlorophyll status. However, the period-specific residual distributions revealed clear differences among T1–T8 (**Figures 6C, D**). The residuals at T3 and T4 were more concentrated around zero, whereas those in the early and late stages were more dispersed, particularly for SPAD. These results indicate that although the model had good overall predictive performance, the reliability of its spatial mapping was clearly constrained by the phenological stage of cotton. Therefore, further period-specific validation was conducted to identify reliable mapping windows for collaborative LAI–SPAD retrieval.

Period-specific validation results showed that model performance differed markedly across T1–T8 (**Figure 6**; **Table 6**). Model accuracy was generally low during the early T1–T2 periods, whereas T3 was the most stable period for collaborative LAI and SPAD estimation, with R^2^, RMSE, and MAE values of 0.86, 0.17, and 0.12 for LAI, respectively, and corresponding values of 0.73, 2.85, and 2.25 for SPAD. T3 simultaneously achieved relatively high R^2^ values and low absolute errors for both targets and was therefore identified as the primary reliable window. The model also maintained good predictive performance at T4, with R^2^ values of 0.71 and 0.65 and RMSE values of 0.27 and 2.82 for LAI and SPAD, respectively. Although the dual-target predictive performance at T4 was lower than that at T3, it still maintained relatively high R^2^ values and low errors and was therefore identified as a supplementary reliable window. Model performance declined further during T5–T6. At T5, LAI retained a certain level of predictive capability (R^2^ = 0.63), whereas the R^2^ for SPAD decreased to 0.45; at T6, the R^2^ values for LAI and SPAD declined to 0.47 and 0.38, respectively, and model accuracy was lowest during T7–T8.

**Table 6:** Period-specific out-of-fold prediction performance of the optimized MIC_union–MTE model.

Period	LAI R^2^	LAI RMSE	LAI MAE	SPAD R^2^	SPAD RMSE	SPAD MAE
T1	0.34	0.46	0.36	0.18	6.02	4.37
T2	0.25	0.47	0.35	0.40	4.29	3.83
T3	0.86	0.17	0.12	0.73	2.85	2.25
T4	0.71	0.27	0.20	0.65	2.82	2.25
T5	0.63	0.31	0.23	0.45	3.37	2.92
T6	0.47	0.41	0.28	0.38	4.30	3.66
T7	0.32	0.49	0.39	0.17	6.15	4.66
T8	0.22	0.53	0.44	0.13	4.85	4.24

In addition, to examine whether the 15-day temporal window affected phenological-period-scaleretrieval performance for LAI and SPAD, a W5 sensitivity analysis was conducted based on the actualfield-survey periods in 2021. W15 retained all 872 site–period records, whereas W5 retained 853 complete records, accounting for 97.8% of all observations; to ensure comparability between the two temporal compositing schemes, both W15 and W5 were evaluated using the same 853 valid plot–period records and identical nested five-fold cross-validation partitions grouped by sampling-site ID. On this common sample set, the overall LAI R^2^ values for W15 and W5 were 0.91 and 0.88, respectively, with corresponding RMSE values of 0.41 and 0.46; the overall SPAD R^2^ values were 0.79 and 0.73, with RMSE values of 4.49 and 5.21, respectively (**Supplementary Table S5**). W15 achieved lower LAI RMSE values in all eight periods and lower SPAD RMSE values inseven periods; shortening the window to 5 days also did not improve predictive accuracy at T3, corresponding to the squaring stage, or T4, corresponding to the early flowering stage, which were specifically highlighted by the reviewer. These results indicate that, under the data conditions of this study, W15 did not reduce retrieval performance at the phenological-period scale. The temporal windows and actual image acquisition dates for W15 and W5 are provided in **Supplementary Tables S1** and **S3**, respectively, and the corresponding period-specific results based on the common set of 853records are presented in **Supplementary Tables S2** and **S4**.

### 3.5 Point-scale external validation using 2025 samples

To evaluate the cross-year transferability of the models, the optimal period-specific models developed for T3, T4, and T6 using the 2021 data were directly applied to independent samples collected during the corresponding periods in 2025 for point-scale external validation.

**Figure 7:**
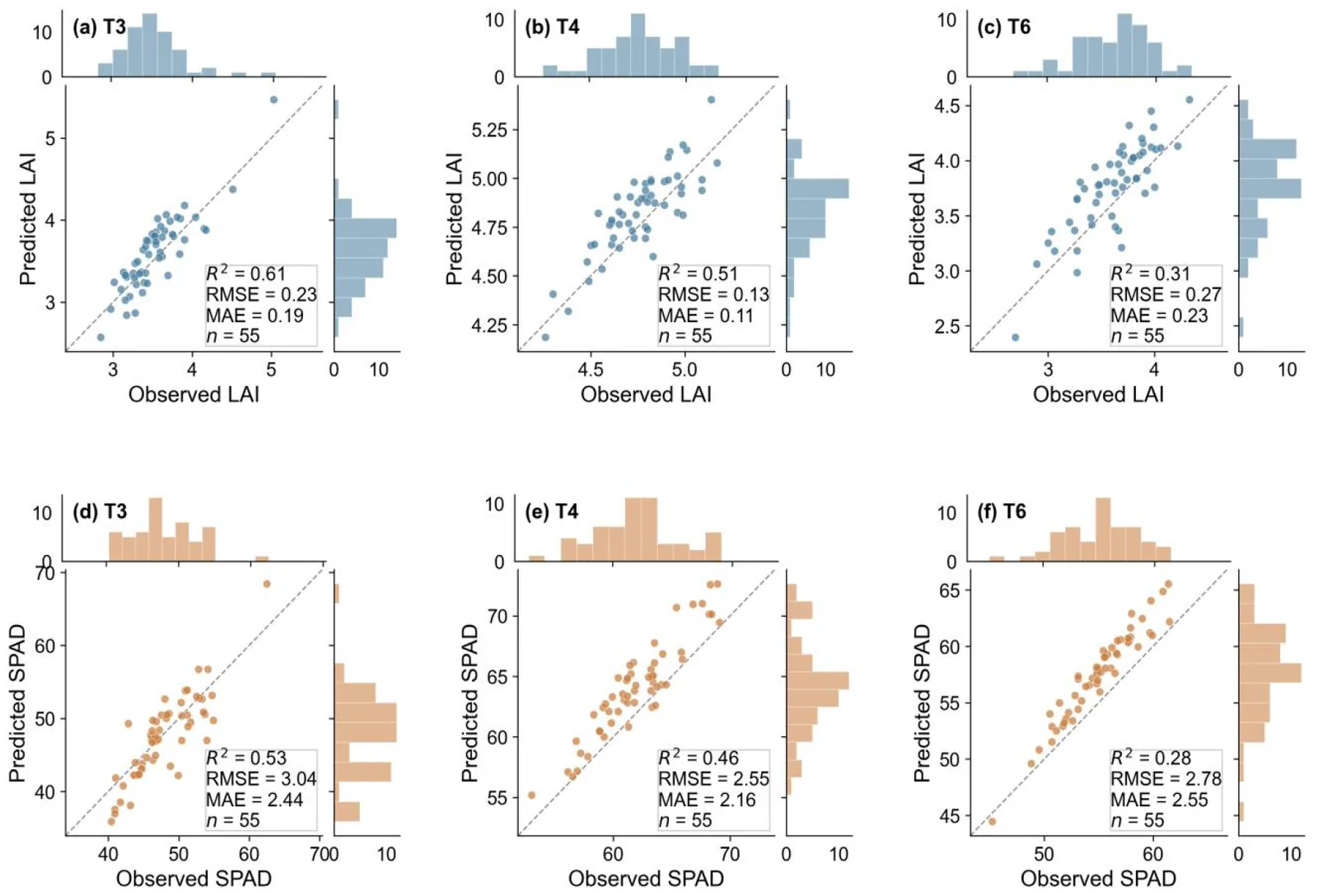
Point-scale external validation of LAI and SPAD predictions using independent 2025 samples: **(a–c)** LAI validation at T3, T4, and T6, respectively, and **(d–f)** SPAD validation at T3, T4, and T6, respectively.

The external validation results showed that the models retained a certain degree of cross-year predictive capability during all three periods, although their performance differed markedly among periods (**Table 7**; **Figure 7**). For LAI, T3 achieved the highest R^2^ (0.61), with RMSE and MAE values of 0.23 and 0.19, respectively; T4 yielded an R^2^ of 0.51 but the lowest RMSE (0.13) and MAE (0.11); predictive performance was comparatively weaker at T6, where R^2^ decreased to 0.31. For SPAD, T3 likewise achieved the highest R^2^ (0.53), whereas T4 had the lowest RMSE (2.55) and MAE (2.16); T6 produced the lowest R^2^, at 0.28.

**Table 7:** Point-scale external validation of the 2021 period-specific MTE models using independent samples collected in 2025.

Period	n	LAI R^2^	LAI RMSE	LAI MAE	LAI MBE	SPAD R^2^	SPAD RMSE	SPAD MAE	SPAD MBE
T3	55	0.61	0.23	0.19	0.07	0.53	3.04	2.44	-0.43
T4	55	0.51	0.13	0.11	0.07	0.46	2.55	2.16	2.07
T6	55	0.31	0.27	0.23	0.15	0.28	2.78	2.55	2.52

MBE denotes mean bias error and was calculated as the mean difference between predicted and observed values. Positive and negative MBE values indicate overall overestimation and underestimation, respectively.

The MBE results further indicated slight overestimation of LAI during all three periods, although the overall systematic bias was small. The MBE for SPAD at T3 was −0.43, indicating an overall bias close to zero; the corresponding MBE values at T4 and T6 were 2.07 and 2.52, respectively, indicating a certain degree of systematic overestimation during these two periods. Considering R^2^, RMSE, MAE, and MBE together, T3 exhibited the most balanced cross-year predictive performance for both targets; T4 showed relatively low absolute prediction errors but a certain positive bias in SPAD prediction; and T6 exhibited comparatively weak external validation performance.

### 3.6 Spatial mapping of LAI and SPAD during reliable phenological windows

**Figure 8:**
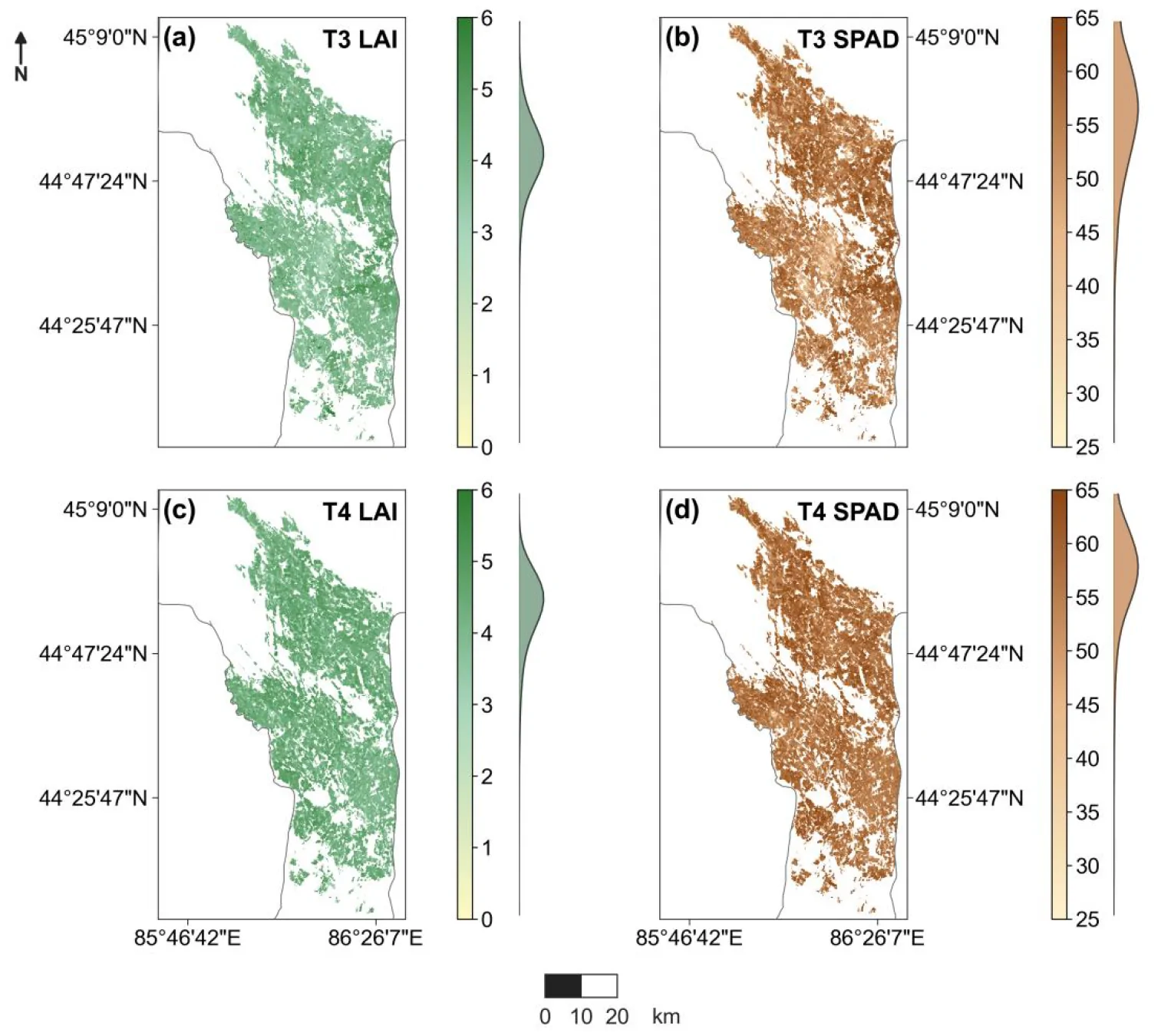
Spatial distribution of mapped LAI and SPAD during reliable phenological windows: **(a)** T3 LAI, **(b)** T3 SPAD, **(c)** T4 LAI, and **(d)** T4 SPAD. Marginal distributions summarize the value range and density of each mapped variable.

Spatial retrieval of cotton-field LAI and SPAD was conducted for the T3 and T4 periods (**Figure 8**). The spatial mapping results showed that the model-estimated LAI and SPAD values at T3 and T4 were generally within relatively high ranges. During T3, the pixel-level mean and median values of LAI were 4.13 and 4.16, respectively, with an interquartile range of 3.81–4.50; during the same period, the mean and median SPAD values were 55.33 and 57.22, respectively, with an interquartile range of 52.58–62.12 (**Table 8**). These results indicate that both canopy structure and chlorophyll status of cotton fields were at relatively high levels during T3, with a comparatively continuous spatial distribution.

**Table 8:** Pixel-level descriptive statistics of mapped LAI and SPAD during reliable phenological windows.

Period	Variable	Mean	Median	SD	CV (%)	P25	P75
T3	LAI	4.13	4.16	0.79	19.11	3.81	4.50
T3	SPAD	55.33	57.22	9.16	16.56	52.58	62.12
T4	LAI	4.43	4.64	0.93	21.04	4.26	4.93
T4	SPAD	57.38	59.24	8.26	14.40	55.02	62.01

At T4, the pixel-level mean values of both LAI and SPAD were higher than those at T3; the mean and median LAI increased to 4.43 and 4.64, respectively, while the mean and median SPAD were 57.38 and 59.24, respectively (**Table 8**). Compared with T3, the spatial variation in LAI at T4 increased slightly, with the CV rising from 19.11% to 21.04%; in contrast, the CV of SPAD decreased from 16.56% to 14.40%, indicating that chlorophyll status became relatively more concentrated at the county scale during the early flowering and boll-setting stage, whereas differences in canopy structure increased. Therefore, T4 can still serve as a supplementary reliable window.

**Figure 9:**
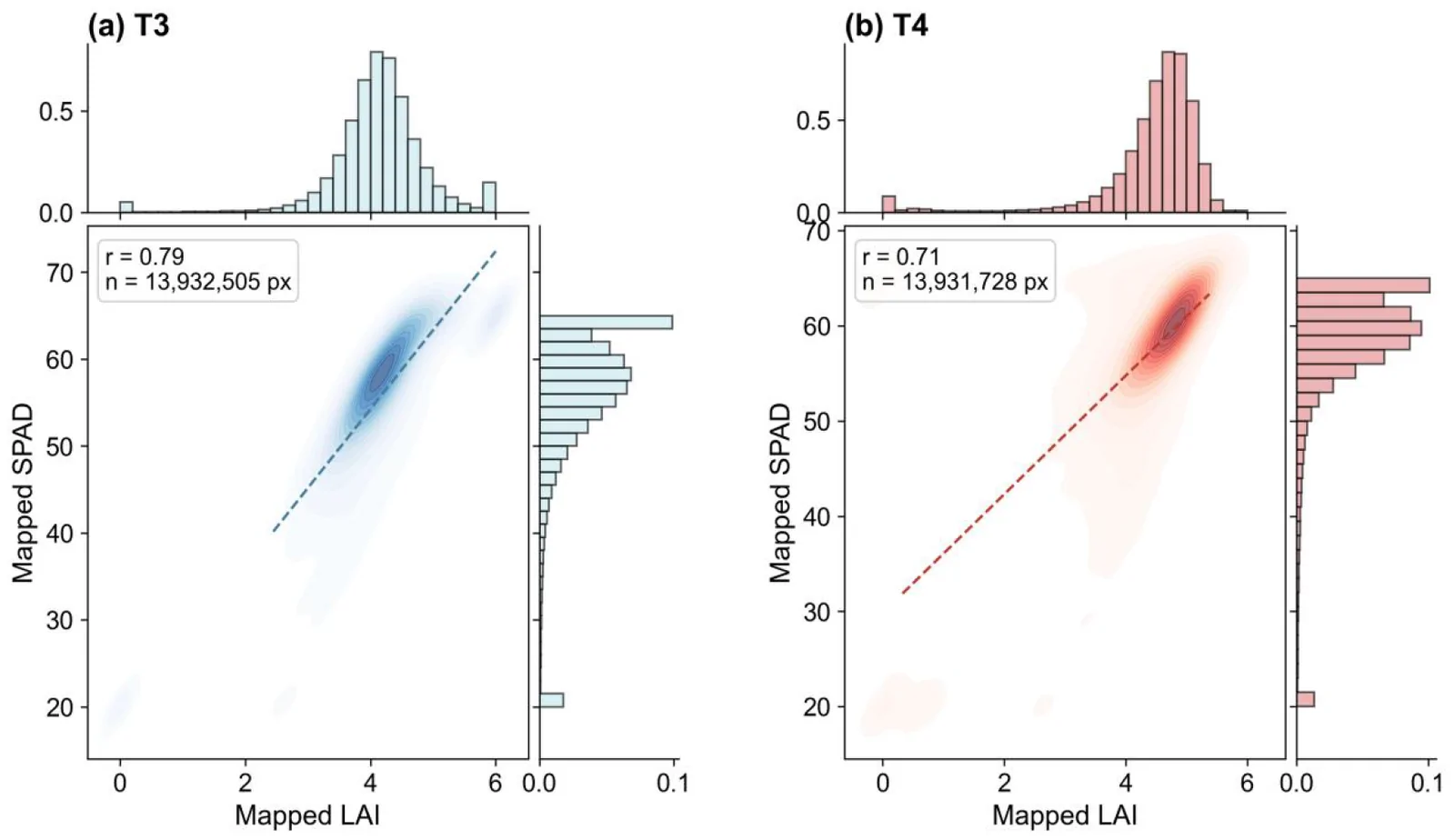
Pixel-level relationship between mapped LAI and SPAD during T3 and T4: **(a)** T3 and **(b)** T4. Scatter-density plots with marginal histograms illustrate the pixel-level association between mapped LAI and SPAD and its variation between the two observation periods.

The pixel-scale LAI–SPAD relationship further revealed a significant positive correlation between model-estimated canopy structure and chlorophyll status within both reliable windows (**Figure 9**). LAI and SPAD exhibited a strong spatial correlation at T3 (Pearson r = 0.79), indicating that the two indicators showed relatively consistent spatial distribution patterns during this period. At T4, LAI and SPAD remained strongly positively correlated, although the correlation coefficient decreased to 0.71, indicating that their spatial correspondence weakened as cotton entered the flowering and boll-setting stage and exhibited a certain degree of stage-specific differentiation.

To ensure the completeness of the mapping results, the spatial retrieval results of LAI and SPAD across all T1–T8 periods are presented in the **Supplementary Materials** (**Supplementary Figure S2**). Given that the period-specific and external validation results indicated lower mapping reliability for T1, T2, and T5–T8 than for T3/T4, the main spatial analysis focused on the two reliable windows, T3 and T4.

## 4 Discussion

### 4.1 Phenology-dependent coordination and differentiation of LAI and SPAD

Both LAI and SPAD in cotton exhibited pronounced phenology-dependent variations, although their stage-specific responses were not fully synchronized. LAI primarily characterizes canopy structure, leaf-area accumulation, and light-interception capacity, and its overall pattern of increasing during the early growth stage, remaining high during the middle stage, and declining during the late stage is consistent with cotton canopy expansion and subsequent structural degradation (Fan et al., 2023; Li et al., 2022). SPAD reflects the relative chlorophyll status of leaves, remaining at a relatively high level during the flowering and boll-setting stage and gradually decreasing during the late growth stage, a pattern consistent with the maintenance of leaf function and subsequent senescence in cotton (Zhou and Yang, 2023).

LAI and SPAD exhibited strong coordination during rapid canopy development and flowering and boll formation, but gradually diverged during the late growth stage (Guo et al., 2010). LAI is more sensitive to leaf abscission, reductions in leaf area, and changes in canopy structure, whereas SPAD primarily reflects the relative chlorophyll status of the remaining functional leaves; consequently, their peak periods and rates of decline are not fully consistent (Zhao et al., 2022). This stage-dependent coordination and differentiation indicate that LAI and SPAD characterize distinct dimensions of canopy structure and leaf physiological status, respectively, and that their combined use can provide more comprehensive information on cotton growth than either indicator alone.

### 4.2 Phenology-dependent spectral basis for joint LAI–SPAD retrieval

Period-specific MIC analysis showed that the spectral features sensitive to LAI and SPAD contained shared spectral information while also exhibiting clear target specificity and temporal variation. NDRE, MTCI, S2REP, and the Sentinel-2 red-edge bands were repeatedly selected across multiple periods, indicating that the red-edge region is an important spectral domain linking canopy structure and chlorophyll status. Red-edge reflectance has been shown to be jointly influenced by chlorophyll absorption, leaf area, leaf inclination angle, and canopy cover; therefore, these features may contain both pigment information related to SPAD and structural information related to LAI (Xing et al., 2022; Zou et al., 2022). Near-infrared features primarily respond to leaf scattering and canopy structure but may become saturated under high-LAI conditions; some shortwave-infrared features may reflect the combined variation in canopy water content, dry matter, soil background, and non-green components (Yue et al., 2022).

The composition of spectral information changed with phenological progression. Stage-dependent changes in canopy cover, leaf area, chlorophyll status, and background components can alter the statistical relationships of red-edge, near-infrared, and shortwave-infrared features with LAI and SPAD (Berger et al., 2021). Therefore, the significance of period-specific MIC screening lies not only in reducing the number of variables but also in adapting to changes in spectral–trait relationships across different phenological stages.

### 4.3 Structural suitability of MTE for joint LAI–SPAD retrieval

MTE achieved the best performance among the six candidate algorithms. This result may be related to the structured constraints imposed by MTE on correlated multi-output tasks. Multi-task learning introduces inductive constraints by sharing partial representations or parameter structures across related tasks, thereby helping to improve the stability of parameter estimation under limited-sample conditions (Caruana, 1997). Specifically, MTE employs elastic-net regularization composed of a cross-task row-sparse L2,1 penalty and an L2-type smoothing penalty, causing the coefficients of the same spectral feature across the LAI and SPAD tasks to be selected or shrunk jointly and thereby forming a shared feature-support structure (Yuan and Lin, 2006; Argyriou et al., 2008). At the same time, the two targets retain their own regression coefficients, allowing shared features to exert different effect strengths on LAI and SPAD.

The suitability of MTE is also related to the substantial information overlap among the spectral variables. The L2-type smooth shrinkage in Elastic Net can stabilize the coefficients of correlated variables, whereas the L1-type constraint provides variable-selection and compression capability, thereby reducing the influence of multicollinearity on parameter estimation (Zou and Hastie, 2005). The optimal model in this study had an l1_ratio of 0.1, indicating that predominantly smooth shrinkage with a relatively weak joint-sparsity component was more suitable for the highly correlated spectral inputs and limited period-specific samples used here. In contrast, models that fit the two targets independently cannot explicitly share task parameters, whereas the mechanisms by which RF and PLS2 utilize multi-output information also lack the joint constraint of “shared feature support–task-specific coefficients” provided by MTE. Therefore, the relative advantage of MTE mainly arises from its balance among task-information sharing, preservation of target-specific differences, and control of multicollinearity.

### 4.4 Reliable phenological windows for structural–physiological mapping of cotton

Period-specific evaluation showed that the reliability of collaborative LAI–SPAD retrieval exhibited clear phenological dependence. T3 corresponded to the late squaring stage and approached the transition to flowering and boll setting; both the 2021 out-of-sample evaluation and the 2025 independent validation showed relatively balanced predictive performance for the two targets, providing direct cross-year evidence for identifying T3 as a reliable window. Similar stage-specific advantages have been reported in remote-sensing studies of cotton LAI and chlorophyll status (Jiang et al., 2025; Lacerda et al., 2025). At T4, the model continued to produce relatively low absolute prediction errors, but R^2^ decreased and SPAD predictions exhibited a certain positive bias, indicating that the characterization of relative differences among sampling sites and the control of cross-year bias were less stable than at T3. Therefore, T4 can still provide supplementary characterization of the spatial LAI–SPAD status during the early flowering and boll-setting stage, although greater attention should be paid to systematic bias in SPAD when applying the model across years.

Independent-year validation further showed that, compared with T3 and T4, the ability of the T6 model to explain variation in both LAI and SPAD was substantially reduced and was accompanied by larger positive biases, indicating that the spectral–trait relationships represented by the model during the middle-to-late flowering and boll-setting stage were more sensitive to interannual variation. Previous studies have shown that changes in canopy condition and background composition can reshape the spectral responses of biophysical traits and affect the stability of empirical models under different growth conditions (Angel and McCabe, 2022; Gao et al., 2022b). Therefore, the selection of reliable windows should be based on stage-specific external validation evidence rather than inferred directly from overall accuracy in a single year or from fixed calendar periods.

### 4.5 Limitations and future perspectives

Although this study identified relatively reliable windows for collaborative LAI–SPAD mapping, several limitations remain. First, the models were primarily based on statistical associations between Sentinel-2 canopy reflectance and field-observed LAI and SPAD values and did not independently separate the mixed spectral effects of canopy structure, chlorophyll absorption, soil background, shadows, and non-green plant organs. Therefore, the associated mechanistic interpretations require further validation using canopy-component observations or radiative transfer simulations. In addition, SPAD is a leaf-scale indicator of relative chlorophyll status; therefore, the retrieval results in this study should be interpreted as spatial representations of relative chlorophyll status at the plot scale rather than as absolute chlorophyll content. Plot-center coordinates were recorded using a handheld GPS receiver, and some plot-to-pixel misregistration may therefore remain when matching the 10 m × 10 m field plots with Sentinel-2–10 m pixels. Although relatively homogeneous canopy areas were selected and field boundaries and other mixed land-cover features were avoided during sampling, this positional uncertainty could still contribute to errors in point-scale spectral extraction and empirical retrieval.

Second, the W15 composite images represent the integrated canopy condition over a specified temporal range within the corresponding phenological stage rather than the instantaneous spectral condition at the time of field sampling. Although the paired W5 analysis showed that W15 produced lower overall out-of-sample errors, 15 days cannot be regarded as a universally optimal biological timescale, and median compositing may still smooth short-term variation within the temporal window. In addition, some field campaigns were not located near the temporal center of their corresponding W15 windows, and the Sentinel-2 acquisitions contributing to the composites were not always distributed symmetrically around the survey dates. Consequently, some temporal mismatch between field observations and composite reflectance may remain, particularly during periods of rapid canopy development, such as the transition from squaring to flowering and boll setting.

Third, predictive performance in the independent 2025 validation declined markedly relative to the 2021 model-development results, indicating that empirical spectral–trait relationships derived from a single year underwent performance degradation when transferred across years. This interannual generalization gap suggests that the models may be sensitive to year-to-year variation in canopy development, environmental background, cotton cultivar, and field management. At the same time, the decline in cross-year performance was clearly period dependent, with T3 and T4 retaining better joint predictive performance than T6. This further supports the identification of T3 as the primary reliable observation window and T4 as a supplementary window. However, this reliability should be interpreted as relative stability within specific phenological stages and validated conditions rather than universal cross-year transferability throughout the entire growing season. In addition, the 2025 point-scale external validation covered only T3, T4, and T6, and sampling-site placement was not strictly stratified by soil type, cotton cultivar, or water and fertilizer management. Future independent validation across additional years, phenological stages, and management conditions is therefore required to further assess model generalizability.

## 5 Conclusions

This study developed a phenology-aware framework for collaborative LAI–SPAD retrieval by integrating period-specific MIC feature selection with multi-task learning. LAI and SPAD shared certain spectral and phenological information while also exhibiting clear stage-specific differences. The MIC_union feature scheme consistently achieved superior joint predictive performance across different multi-output models, while MTE provided the best overall performance among the six candidate regression algorithms. Based on the period-specific out-of-sample evaluation in 2021 and independent point-scale validation at T3, T4, and T6 in 2025, model performance exhibited clear phenological dependence, supporting T3 as the primary window for collaborative mapping and T4 as a supplementary window. Overall, the integration of phenological stratification, shared and target-specific feature organization, and multi-task learning provides a technical approach for the collaborative retrieval of cotton canopy structure and relative chlorophyll status during key growth stages and may inform the selection of observation periods for cotton growth monitoring and precision management.

## Data Availability

The original contributions presented in the study are included in the article/**Supplementary Material**. Further inquiries can be directed to the corresponding author.
