# Supplementary material for: Phenology-aware collaborative retrieval of structural and physiological cotton traits from Sentinel-2 imagery using multi-task learning
Source: Front Plant Sci. 2026 Aug 25;17:1928307. doi: 10.3389/fpls.2026.1928307 (PMC13550183; doi:10.3389/fpls.2026.1928307)
Supplement: Supplementary file 1 [file SupplementaryFile1.pdf]

Supplementary Material

Supplementary Table S1. Field campaigns, 15-day median-compositing windows (W15), and actual retained Sentinel-2 acquisition dates in 2021.

Period	Field campaign	W15 window	Actual retained S2 dates
T1	2021-06-02–2021-06-04	2021-06-01–2021-06-15	2021-06-02, 2021-06-07, 2021-06-10, 2021-06-12, 2021-06-15
T2	2021-06-18–2021-06-21	2021-06-16–2021-06-30	2021-06-20, 2021-06-27, 2021-06-30
T3	2021-07-02–2021-07-05	2021-07-01–2021-07-15	2021-07-02, 2021-07-05, 2021-07-10, 2021-07-12, 2021-07-15
T4	2021-07-18–2021-07-21	2021-07-16–2021-07-30	2021-07-20, 2021-07-25, 2021-07-27
T5	2021-08-03–2021-08-05	2021-07-31–2021-08-14	2021-08-01, 2021-08-04, 2021-08-06, 2021-08-09, 2021-08-11
T6	2021-08-20–2021-08-24	2021-08-15–2021-08-29	2021-08-16, 2021-08-19, 2021-08-21, 2021-08-24, 2021-08-29
T7	2021-09-05–2021-09-07	2021-08-30–2021-09-13	2021-08-31, 2021-09-03, 2021-09-05, 2021-09-08, 2021-09-13
T8	2021-09-20–2021-09-24	2021-09-14–2021-09-28	2021-09-15, 2021-09-18, 2021-09-20, 2021-09-23, 2021-09-25, 2021-09-28

Supplementary Table S2. Period-specific out-of-fold performance of the MIC_union–MTE model under five-fold GroupKFold using W15 composites and the identical matched sample set.

Period	n	LAI_R^2	LAI_RMSE	LAI_MAE	SPAD_R^2	SPAD_RMSE	SPAD_MAE
T1	109	0.34	0.46	0.36	0.18	6.02	4.37
T2	97	0.20	0.48	0.34	0.39	4.19	3.70
T3	106	0.88	0.15	0.11	0.75	2.73	2.18
T4	109	0.71	0.27	0.20	0.65	2.81	2.24
T5	108	0.63	0.32	0.23	0.46	3.37	2.92
T6	109	0.47	0.41	0.28	0.38	4.30	3.66
T7	109	0.32	0.49	0.39	0.17	6.15	4.66
T8	106	0.21	0.53	0.45	0.13	4.90	4.27

Supplementary Table S3. Field campaigns, field-aligned 5-day median-compositing windows (W5), and actual retained Sentinel-2 acquisition dates in 2021.

Period	Field campaign	W5 window	Actual retained S2 dates
T1	2021-06-02–06-04	2021-05-31–06-04	2021-05-31, 2021-06-02
T2	2021-06-18–06-21	2021-06-17–06-21	2021-06-20
T3	2021-07-02–07-05	2021-07-01–07-05	2021-07-02, 2021-07-05
T4	2021-07-18–07-21	2021-07-17–07-21	2021-07-20
T5	2021-08-03–08-05	2021-08-01–08-05	2021-08-01, 2021-08-04
T6	2021-08-20–08-24	2021-08-20–08-24	2021-08-21, 2021-08-24
T7	2021-09-05–09-07	2021-09-03–09-07	2021-09-03, 2021-09-05
T8	2021-09-20–09-24	2021-09-20–09-24	2021-09-20, 2021-09-23

Supplementary Table S4. Period-specific out-of-fold performance of the MIC_union–MTE model under five-fold GroupKFold using W5 composites and the identical matched sample set.

Period	n	LAI_R²	LAI_RMSE	LAI_MAE	SPAD_R²	SPAD_RMSE	SPAD_MAE
T1	109	0.08	0.54	0.45	-0.30	7.57	5.66
T2	97	-0.03	0.54	0.41	0.12	5.03	4.26
T3	106	0.29	0.37	0.29	0.31	4.56	3.48
T4	109	0.68	0.28	0.21	0.46	3.50	2.75
T5	108	0.41	0.39	0.29	0.30	3.83	3.11
T6	109	0.41	0.43	0.30	0.29	4.62	3.84
T7	109	0.24	0.52	0.41	0.20	6.05	4.50
T8	106	0.19	0.54	0.45	-0.04	5.34	4.37

Supplementary Table S5. Paired sensitivity comparison of out-of-fold LAI and SPAD retrieval performance between the 15-day (W15) and field-aligned 5-day (W5) median-compositing schemes using the identical matched sample set (n = 853).

Compositing scheme	n	LAI_R²	LAI_RMSE	LAI_MAE	SPAD_R²	SPAD_RMSE	SPAD_MAE
W15	853	0.91	0.41	0.29	0.79	4.49	3.50
W5	853	0.88	0.46	0.35	0.73	5.21	3.99

Supplementary Table S6. Final MIC_union feature sets for each observation period.

Period	Number of features	Features
T1	11	MTCI, B8, GNDVI, NDRE, S2REP, TCARI/OSAVI, B7, CIgreen, B11, NDMI, CIrededge
T2	11	S2REP, B8A, MTCI, NIR_v, B8, B6, B7, NDTI, NDRE, B5, CIrededge
T3	12	B7, NIR_v, OSAVI, NDVI, NDMI, MCARI, NDRE, B8A, S2REP, B6, NDTI, GNDVI
T4	11	NDRE, GNDVI, EVI, MTCI, B8A, NDVI, OSAVI, B6, S2REP, B8, NDMI
T5	5	NDRE, GNDVI, OSAVI, MTCI, S2REP
T6	3	NDRE, S2REP, MTCI
T7	10	MTCI, MSAVI, S2REP, NDRE, B8, B8A, NDMI, B7, B12, NDTI
T8	5	B8, MTCI, MCARI, SAVI, S2REP

Note: A fixed MIC threshold of 0.30 was applied to both LAI and SPAD across all observation periods. The number of features shown represents the final MIC_union feature set after target-specific screening and redundancy removal.

Supplementary Table S7. Model implementation, fixed settings, hyperparameter search spaces, and selected configurations for joint LAI–SPAD retrieval.

Model	Implementation /output strategy	Fixed settings	Hyperparameter search space	Selected hyperparameters
MTE	Native multi-output LAI–SPAD model	max_iter=8000; fit_intercept=True; random_state=42	alpha : {0.0001, 0.001, 0.01, 0.1, 1.0, 10.0}; l1_ratio : {0.1, 0.3, 0.5, 0.7, 0.9}	alpha=0.01; l1_ratio=0.1
RF	Native multi-output tree-based model	random_state=42; n_jobs=-1	n_estimators=200,300,500; max_features='sqrt',0.5,0.7; min_samples_leaf=1,2,3,5; max_depth=None,10,20	n_estimators=500; max_features=0.7; min_samples_leaf=2; max_depth=10
PLS2	Native multi-output LAI–SPAD model	scale=False	n_components=1–min(5,p)	n_components = min(5, p) (base selected value = 5)
XGBoost	MultiOutputRegressor wrapping XGBRegressor	random_state=42; n_jobs=-1; verbosity=0	n_estimators=200,300,500; max_depth=3,5,7; learning_rate=0.01,0.05,0.1; subsample=0.7,0.85,1.0; colsample_bytree=0.7,0.85,1.0	n_estimators=500; max_depth=3; learning_rate=0.01; subsample=0.85; colsample_bytree=0.7
LightGBM	MultiOutputRegressor wrapping LGBMRegressor	random_state=42; n_jobs=-1; verbose=-1	n_estimators=200,300,500; max_depth=4,6,8; learning_rate=0.01,0.05,0.1; num_leaves=15,31,63; subsample=0.7,0.85,1.0; colsample_bytree=0.7,0.85,1.0; reg_lambda=0,0.1,1.0	n_estimators=300; max_depth=8; learning_rate=0.01; num_leaves=31; subsample=0.7; colsample_bytree=0.85; reg_lambda=0.1
SVR-RBF	MultiOutputRegressor wrapping SVR	kernel=rbf; shrinking=True; tol=1e-3; cache_size=500; n_jobs=-1 in MultiOutputRegressor	C=0.1,1,10,100; gamma=scale,0.001,0.01,0.1; epsilon=0.01,0.05,0.1,0.5	C=100; gamma=0.001; epsilon=0.1

Note: Grid search was used for MTE, RF, PLS2, and SVR-RBF, whereas XGBoost and LightGBM used randomized search with 50 candidate combinations. The selected configurations were fixed across T1–T8.

Supplementary Table S8. Sensitivity of LAI–SPAD retrieval performance to feature-input schemes under PLS2 and Random Forest models.

Model	Feature scheme	LAI R2	LAI RMSE	LAI MAE	SPAD R2	SPAD RMSE	SPAD MAE	JNE
PLS2	MIC_union	0.90	0.42	0.31	0.79	4.51	3.56	0.08
PLS2	MIC_shared	0.89	0.44	0.33	0.77	4.82	3.82	0.09
PLS2	All_features	0.90	0.43	0.32	0.76	4.90	3.61	0.09
RF	MIC_union	0.89	0.43	0.32	0.77	4.82	3.71	0.08
RF	MIC_shared	0.89	0.45	0.34	0.76	4.94	3.80	0.09
RF	All_features	0.89	0.44	0.32	0.77	4.77	3.68	0.09

Supplementary Figure S1. Period-specific MIC_union feature networks linking Sentinel-2 spectral features with LAI and SPAD from T1 to T8.

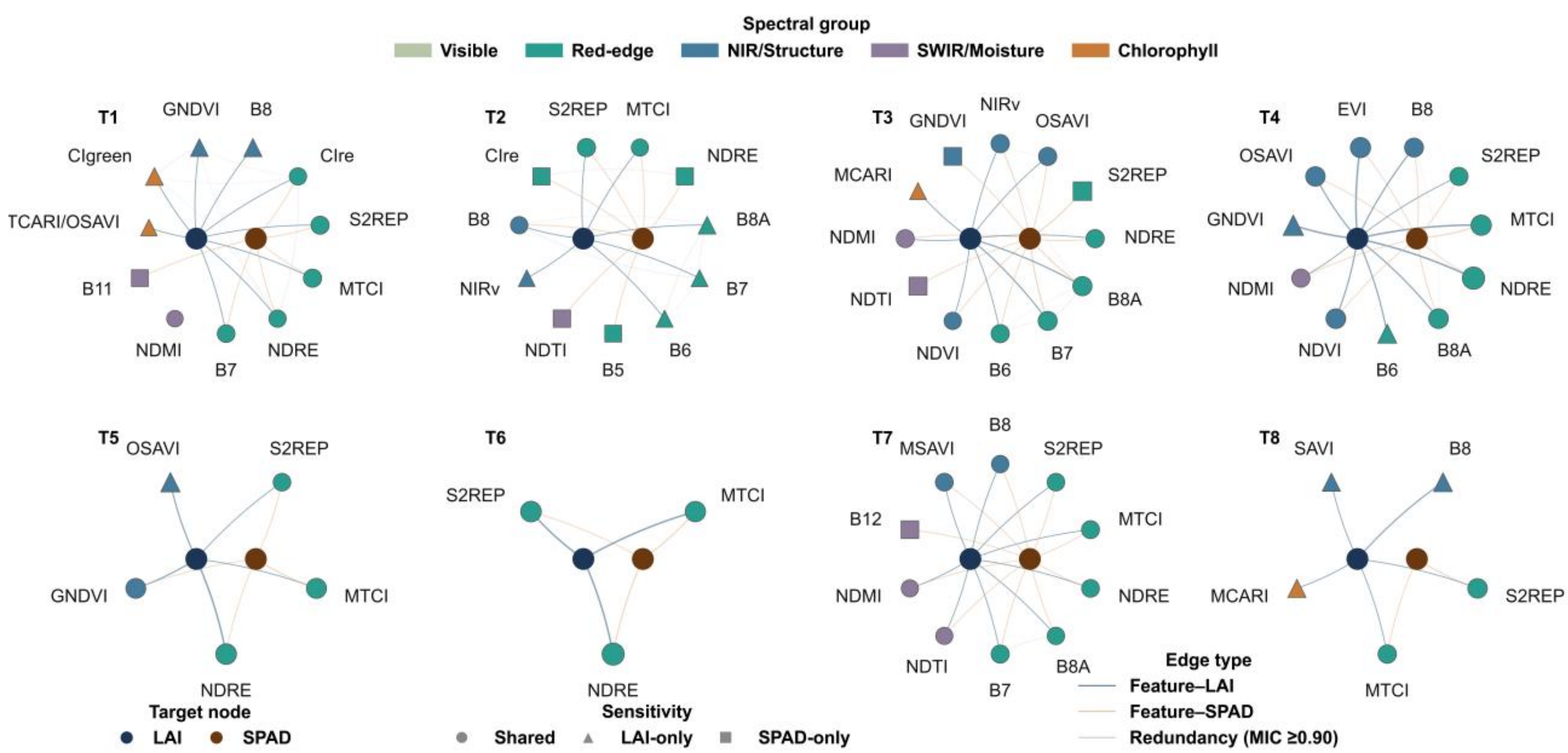

Supplementary Figure S2. All-period spatial maps of LAI and SPAD from T1 to T8. These maps are provided for completeness; interpretation should prioritize the reliable T3–T4 mapping windows identified by period-specific validation.

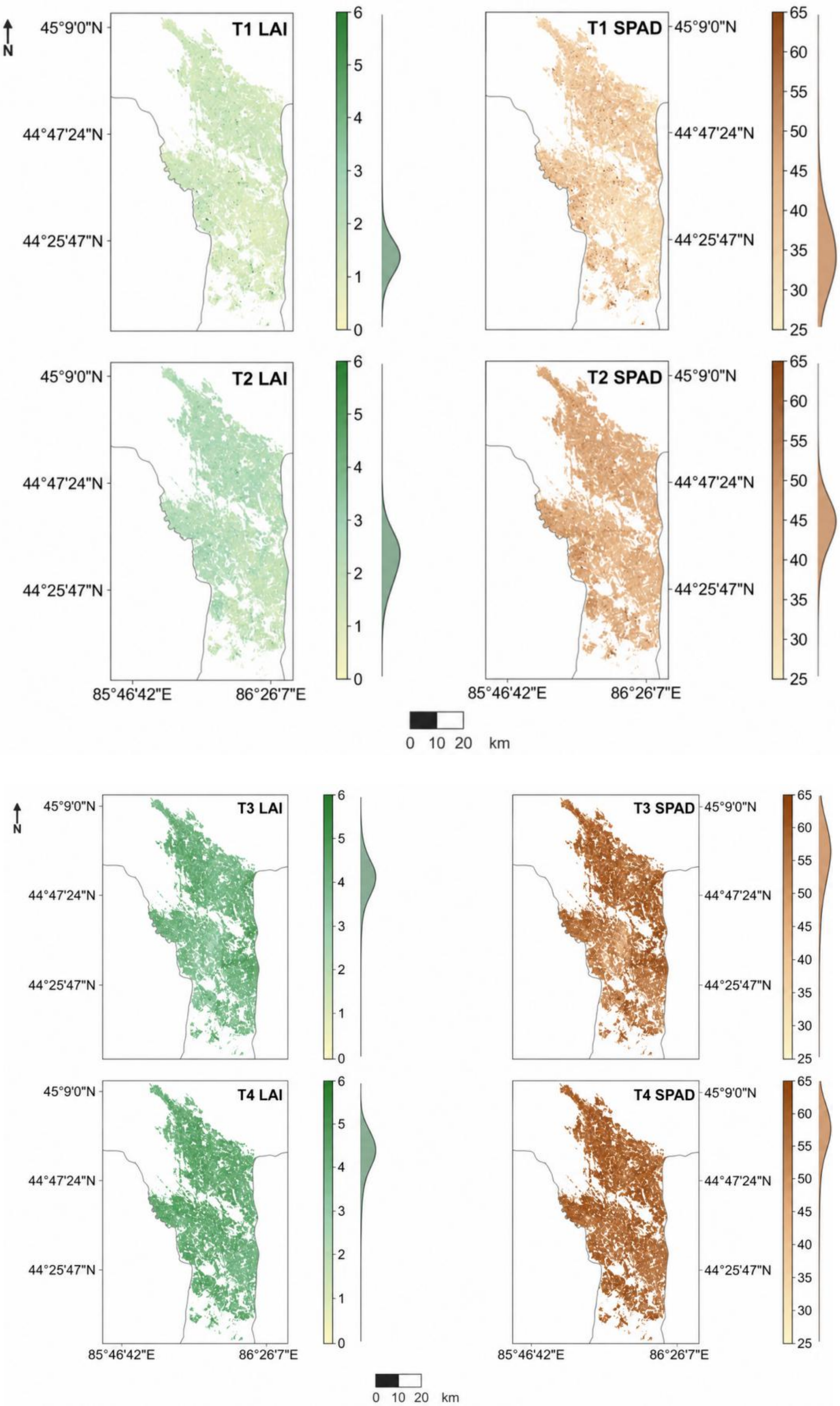

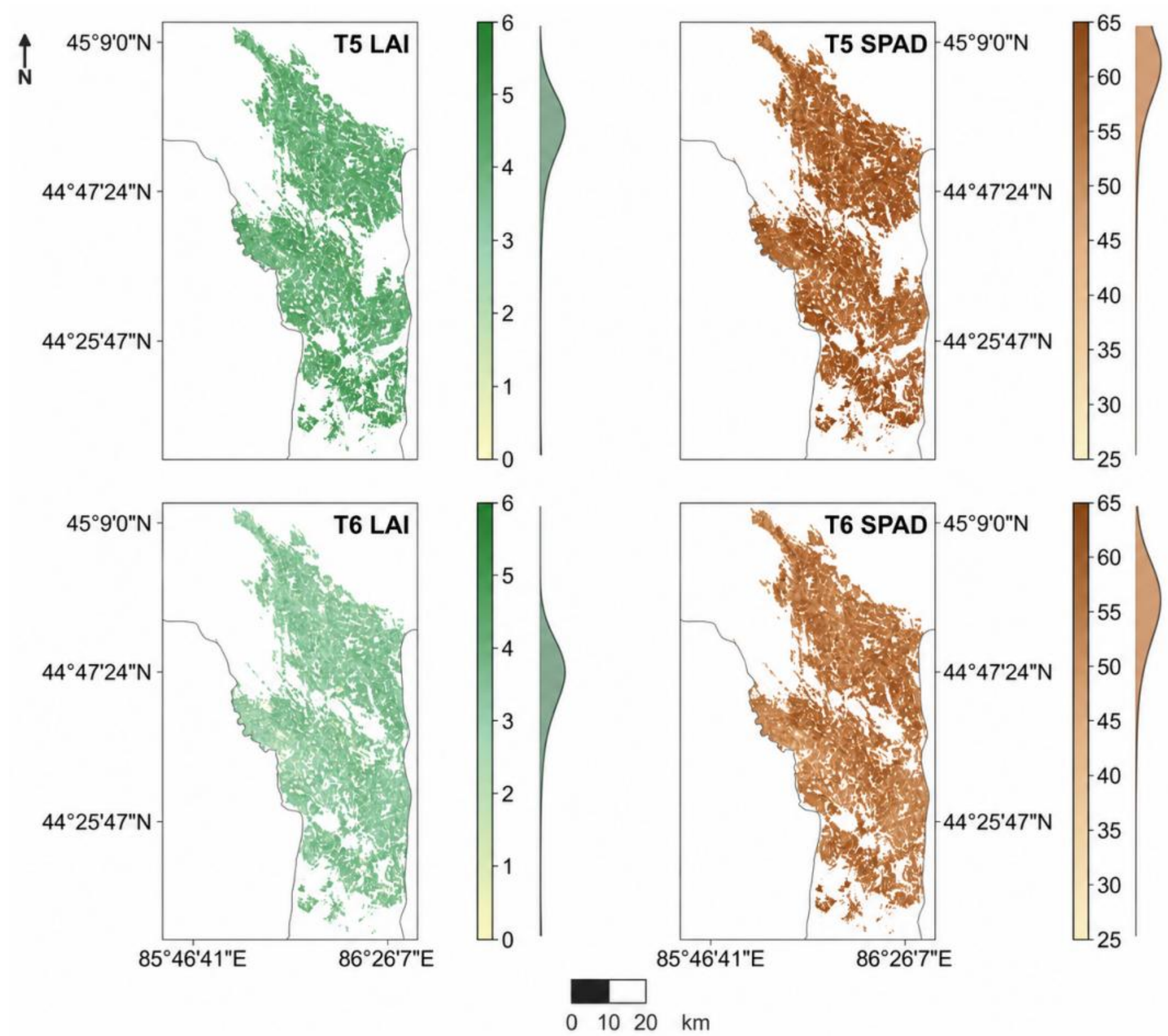
T5 LAI
T5 SPAD
T6 LAI
T6 SPAD
45°9'0"N
44°47'24"N
44°25'47"N
85°46'41"E
86°26'7"E
N
0 10 20 km

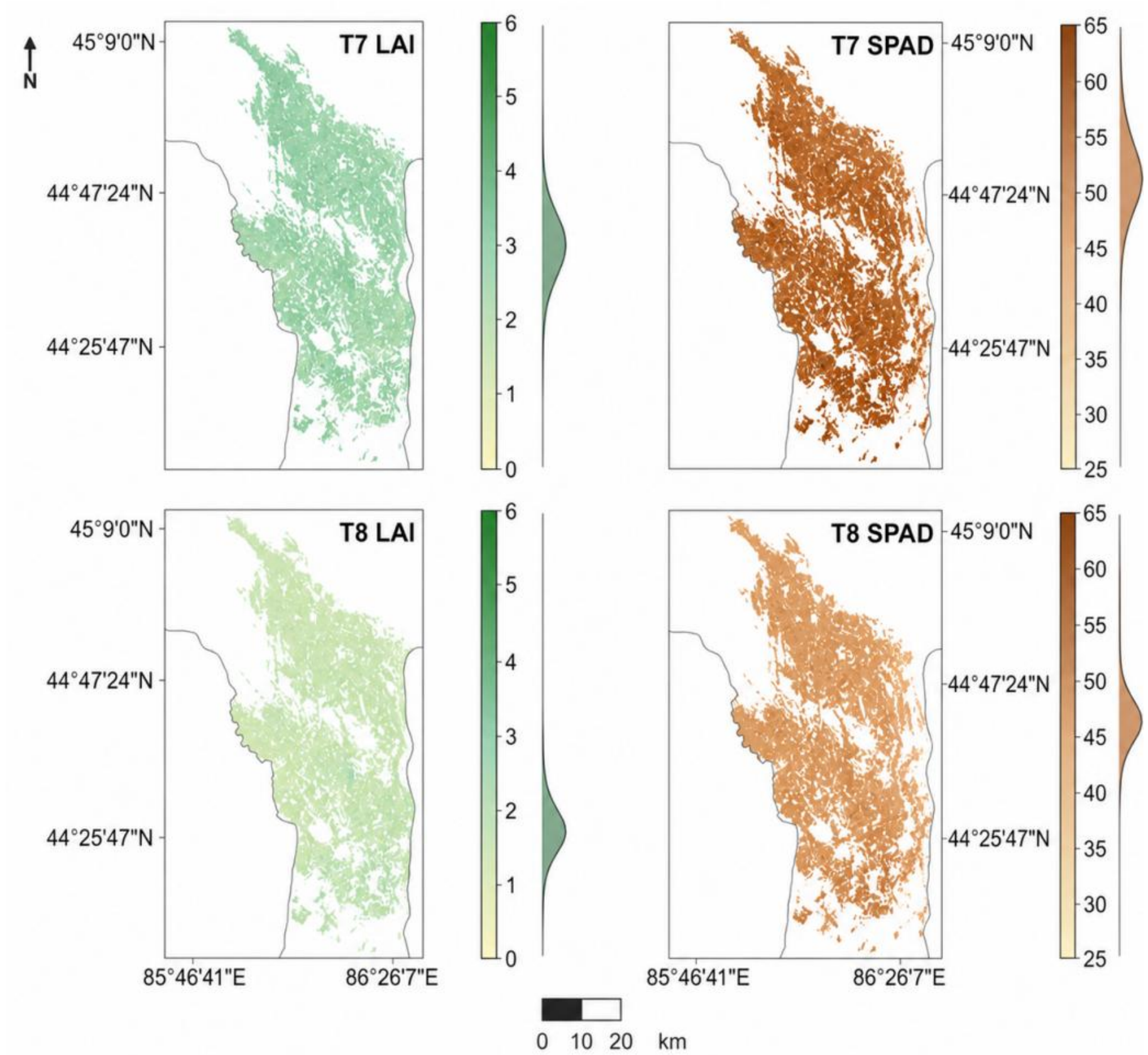
T7 LAI
T7 SPAD
T8 LAI
T8 SPAD
45°9'0"N
44°47'24"N
44°25'47"N
85°46'41"E
86°26'7"E
N
0 10 20 km
